# Single-cell RNA sequencing and spatial transcriptomics of bladder Ewing sarcoma

**DOI:** 10.1016/j.isci.2024.110921

**Published:** 2024-09-11

**Authors:** Weipu Mao, Kangjie Xu, Keyi Wang, Houliang Zhang, Jie Ji, Jiang Geng, Si Sun, Chaoming Gu, Atrayee Bhattacharya, Cheng Fang, Tao Tao, Ming Chen, Jianping Wu, Shuqiu Chen, Chao Sun, Bin Xu

**Affiliations:** 1 Department of Urology, Affiliated Zhongda Hospital of Southeast University, Nanjing 210009, China; 2 Central Laboratory Department, Binhai County People’s Hospital, Yancheng 224000, China; 3 Department of Urology, Shanghai Tenth People’s Hospital, School of Medicine, Tongji University, Shanghai 200072, China; 4 Department of Medical Oncology, Dana-Farber Cancer Institute, Harvard Medical School, Boston, MA, USA; 5 Department of Urology, The First Affiliated Hospital of USTC, Division of Life Sciences and Medicine, University of Science and Technology of China, Hefei 230001, China

**Keywords:** molecular biology, cancer, transcriptomics

## Abstract

Bladder Ewing sarcoma/primitive neuroectodermal tumor (bladder ES/PNET) is a rare and highly malignant tumor associated with a poor prognosis, yet its underlying mechanisms remain poorly understood. Here, we employed a combination of single-cell RNA sequencing (scRNA-seq), spatial transcriptomics (ST), and functional analyses to delve into the pathogenesis of bladder ES/PNET. The investigation revealed the presence of specialized types of epithelial cells (referred to as bladder ES-Epi) and mast cells (referred to as bladder ES-Mast) within bladder ES/PNET in comparison to urothelial carcinoma. Notably, TNFRSF12A exhibited significant upregulation in bladder ES/PNET. Furthermore, mast cells possessed the ability to activate epithelial cells through the TNFSF12-TNFRSF12A ligand-receptor signaling pattern. In addition, Enavatuzumab can significantly inhibit the migratory ability of the Ewing sarcoma cell line RD-ES. This groundbreaking study provides unprecedented mechanistic insights into the progression of bladder ES/PNET and introduces a potential therapeutic avenue for treating this challenging malignancy.

## Highlights

• Bladder ES-Epi and bladder ES-Mast were the specialized cell types of bladder ES/PNET
• Mast cells activate the epithelial cells through TNFSF12-TNFRSF12A ligand-receptor
• Enavatuzumab inhibited the migratory ability of the Ewing sarcoma RD-ES cell line

## Graphical abstract

**Figure:**
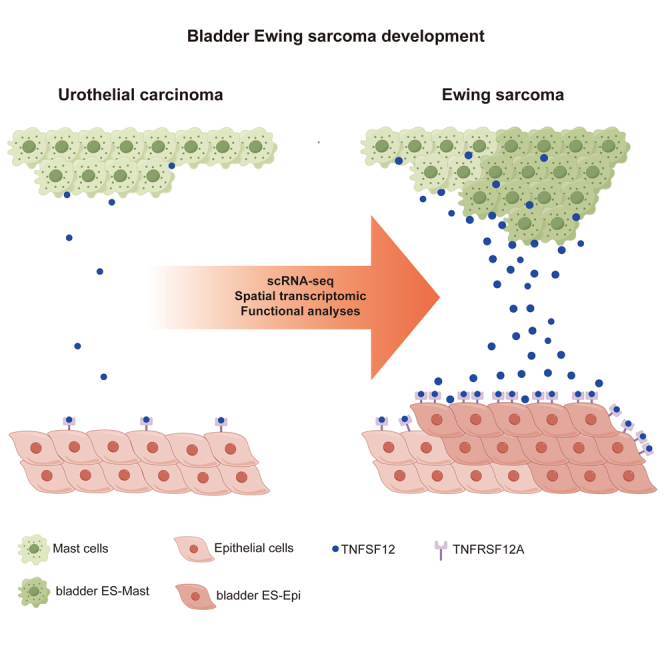

## Introduction

The Ewing family of tumors (EFTs) represents a category of malignant small round-cell tumors characterized by a translocation involving the EWSR1 gene on chromosome 22.^1^ It is the second most prevalent bone tumor among children and adolescents, typically diagnosed during the early twenties, and it demonstrates a high degree of invasiveness.^2^^,^^3^ Primitive neuroectodermal tumor (PNET), classified as a member of the three major types of peripheral EFTs, exhibits highly aggressive behavior.^4^ Primary Ewing sarcoma/PNET (ES/PNET) occurring in the bladder is exceedingly rare, with only a few reported cases and small-scale series.^5^^,^^6^^,^^7^ Despite the recommendation of modern multimodal treatment approaches such as surgery, adjuvant chemotherapy, and radiation therapy, there remains a lack of standardized management and treatment guidelines specifically tailored for bladder ES/PNET.^3^^,^^8^ The prognosis associated with ES/PNET is extremely poor, with an overall survival rate of less than 1 year predominantly due to recurrence or metastasis.^9^ Consequently, understanding the molecular mechanisms driving bladder ES/PNET progression and identifying novel molecular markers and treatment targets hold significant clinical importance.

Single-cell RNA sequencing (scRNA-seq) is a potent technology that enhances bulk RNA-seq by furnishing transcriptional insights at the single-cell level.^10^ scRNA-seq can be used to unravel gene expression patterns of individual cells within tissues, thus delving into cellular heterogeneity and differentiation.^11^ However, scRNA-seq necessitates the dissociation of organs and tissues into single-cell suspensions, inevitably leading to the loss of spatial localization information.^12^^,^^13^ By contrast, spatial transcriptomics (ST) has found widespread application in studying the cancer microenvironment, serving as an emerging tool that conserves spatial distribution information of gene expression profiles. ST enables the visualization and quantitative analysis of tissue slices, a feature absent in traditional scRNA-seq methods.^11^^,^^14^^,^^15^ scRNA-seq and ST offer complementary strengths: while they systematically identify cellular subtypes within tissues, they also elucidate spatial positions, significantly broadening research methodologies across various disease domains.^16^ Recently, Shi et al.^17^ employed a combination of scRNA-seq and ST to scrutinize recurrent bladder urothelial carcinoma (BLCA), unveiling substantial tumor heterogeneity and revealing intricate interactions between epithelial cells and fibroblasts. To decipher the tissue structure and molecular landscape of bladder ES/PNET, the approach integrates scRNA-seq with ST, aiming to explore intercellular communication and potential progression mechanisms across multiple levels and dimensions.

The study employed scRNA-seq, ST, and comprehensive functional analysis techniques to delve deeply into bladder ES/PNET and BLCA tissues alongside matched normal bladder tissues. The objective of this study was to quantitatively depict gene expression at the single-cell level, pinpoint specific clusters with distinct biological effects, visualize spatial information of various cell subtypes, and demonstrate the *in-situ* intercellular communication network. The findings shed light on the molecular mechanisms underlying bladder ES/PNET progression by capturing ligand-receptor signaling transduction in intercellular interactions.

## Results

### Medical history of bladder ES/PNET patient

**Figure 1:**
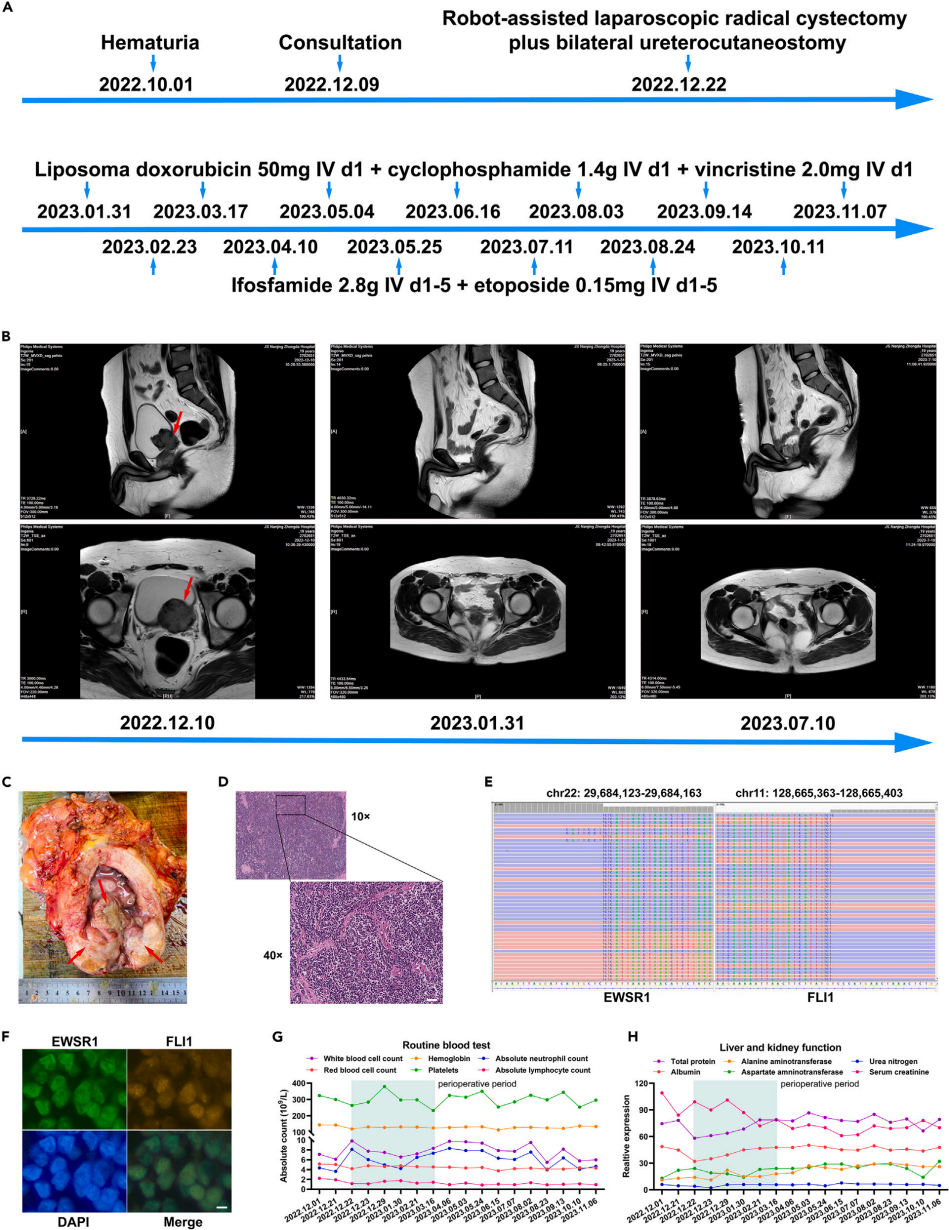
The medical history of bladder ES/PNET patient (A) The patient’s treatment history. (B) MRI assessments at preoperative, 1 month, and 6 months postoperatively. (C) Radical cystoprostatectomy specimen. (D) Small round cells in rosette-like formations on H&E staining (Scale bars, 100 μm). (E) Next-generation sequencing results showing break point of EWSRI-FLI1 fusion. (F) EWSRI-FLI1 gene fusion was detected by fluorescence *in situ* hybridization (FISH) (Scale bars, 20 μm). (G) Changes in routine blood indices throughout the patient’s treatment. (H) Changes in liver and kidney indices throughout the patient’s treatment.

A 19-year-old male patient diagnosed with bladder ES/PNET was referred to our hospital on December 9, 2022, due to experiencing hematuria for a continuous period of 2 months (Figure 1A). Before the consultation, the patient underwent a cystoscopic biopsy at a local hospital, which revealed preliminary bladder ES/PNET pathology. Following comprehensive preoperative examinations involving computed tomography and MRI scans, medical professionals detected a tumor measuring approximately 4.5 cm × 4.5 cm × 3 cm on the left wall of the bladder (Figure 1B). Subsequently, on December 22, 2022, the patient underwent robot-assisted laparoscopic radical cystectomy along with bilateral ureterocutaneostomy. Pathological examination confirmed the presence of bladder ES/PNET at a pathological stage of T3N0M0 (Figures 1C and 1D). Additionally, the next-generation sequencing (NGS) analysis of the specimen revealed a gene rearrangement of EWSR1:FLI1 (Figure 1E), which was further confirmed through fluorescence *in situ* hybridization (FISH) experiments, demonstrating the fusion of the EWSR1 and FLI1 genes (Figure 1F). Between January 31, 2023 and November 20, 2023, the patient underwent alternating chemotherapy regimens, receiving seven cycles of liposome doxorubicin, cyclophosphamide, and vincristine (VAC), along with six cycles of ifosfamide and etoposide (IE) (Figure 1A). Follow-up examinations, including blood routine, liver and kidney function tests (Figures 1G and 1H), and MRI assessments at 1 month and 6 months postoperatively (Figure 1B), indicated the patient’s relatively stable condition.

### Distinct cellular clusters in paired bladder ES/PNET and BLCA identified using scRNA-seq

**Figure 2:**
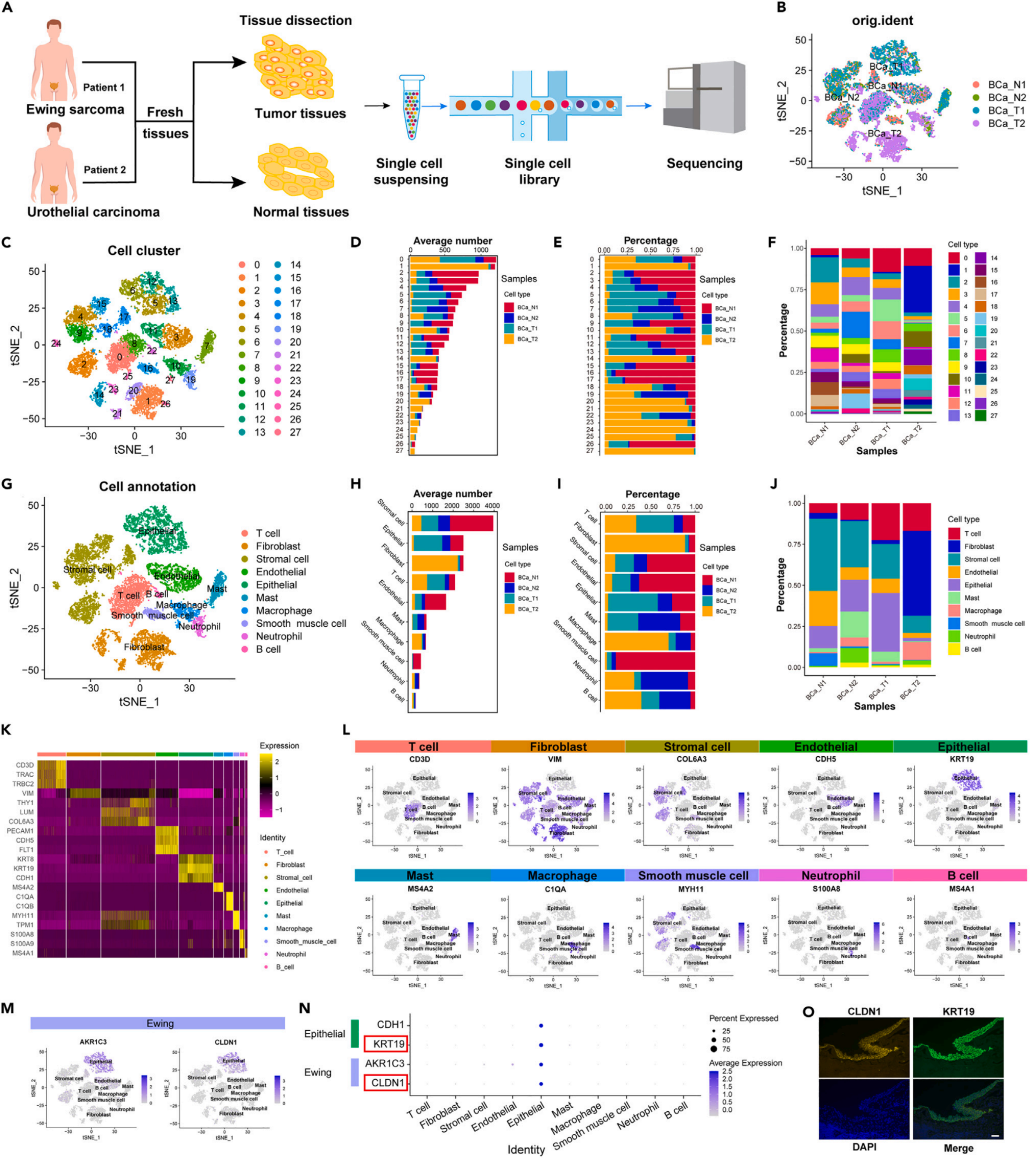
Different cell types in bladder ES/PNET and BLCA identified by scRNA-seq (A) Graphic overview of the design diagram for scRNA-seq. (B) t-SNE plots of 15,037 cells from four samples. (C) t-SNE plots of the 28 clusters. (D–F) Number (D) and percentage (E and F) of cells in 4 samples for 28 clusters. (G) t-SNE plots of the 10 cell types. (H–J) Number (H) and percentage (I and J) of cells in 4 samples for 10 cell types. (K) Heatmap of canonical marker gene expression across 10 major cell types. (L) Feature plots showing the marker gene expressions across the 10 cell types. (M) Feature plots showing the Ewing sarcoma marker gene expressions. (N) Dot plot of the epithelial and Ewing sarcoma marker genes. (O) Immunofluorescence plot of the epithelial and Ewing sarcoma marker genes (Scale bars, 100 μm).

To investigate potential differences in cellular clusters between bladder ES/PNET and BLCA, paired primary tumor and normal tissues were collected for 10× Genomics Chromium scRNA-seq (Figure 2A). After meticulous filtering, a total of 15,037 cells were identified from four samples: BCa_N1, BCa_N2, BCa_T1 (bladder ES/PNET), and BCa_T2 (BLCA) (Figure 2B). Upon integrating the transcriptional data of all cells, a low-resolution t-distributed stochastic neighborhood embedding (t-SNE) clustering technique was initially employed, resulting in a two-dimensional map displaying 28 clusters across the four samples (Figure 2C). This map illustrated the percentages and cell numbers within various clusters for each sample (Figures 2D–2F). Subsequent classification of the 28 cell clusters using well-known markers revealed 10 major cell types (Figure 2G), including T cells marked by CD3D, TRAC, and TRBC2, fibroblasts marked by VIM, stromal cells marked by THY1, LUM, and COL6A3, endothelial cells marked by PECAM1, CDH15, and FLT1, epithelial cells marked by KRT8, KRT19, and CDH1, mast cells marked by MS4A2, macrophages marked by C1QA and C1QB, smooth muscle cells marked by MYH11 and TPM1, neutrophils marked by S100A8 and S100A9, and B cells marked by MS4A1 (Figures 2K and 2L). A comparative analysis against normal tissues revealed increased T cell presence in both bladder ES/PNET and BLCA tissues, whereas endothelial and smooth muscle cells showed decreased presence. Epithelial and mast cells demonstrated increased presence in bladder ES/PNET tumor tissues but decreased presence in BLCA tissues (Figures 2H–2J). Aynaud et al.^18^ reported that AKR1C3 and CLDN1 as two marker genes for Ewing sarcoma. Consequently, the expression of AKR1C3 and CLDN1 was examined in all cell types, and their predominant expression was found in epithelial cells (Figure 2M). Furthermore, these cells exhibited high expression levels of both epithelial cell marker genes and Ewing sarcoma marker genes, indicating that the detected epithelial cells possess Ewing sarcoma characteristics (Figure 2N). Additionally, immunofluorescence analysis was performed to study the co-localization of the epithelial cell marker gene KRT19 and the Ewing sarcoma marker gene CLDN1 in bladder epithelium (Figure 2O).

### Heterogeneity exists between different cell types

**Figure 3:**
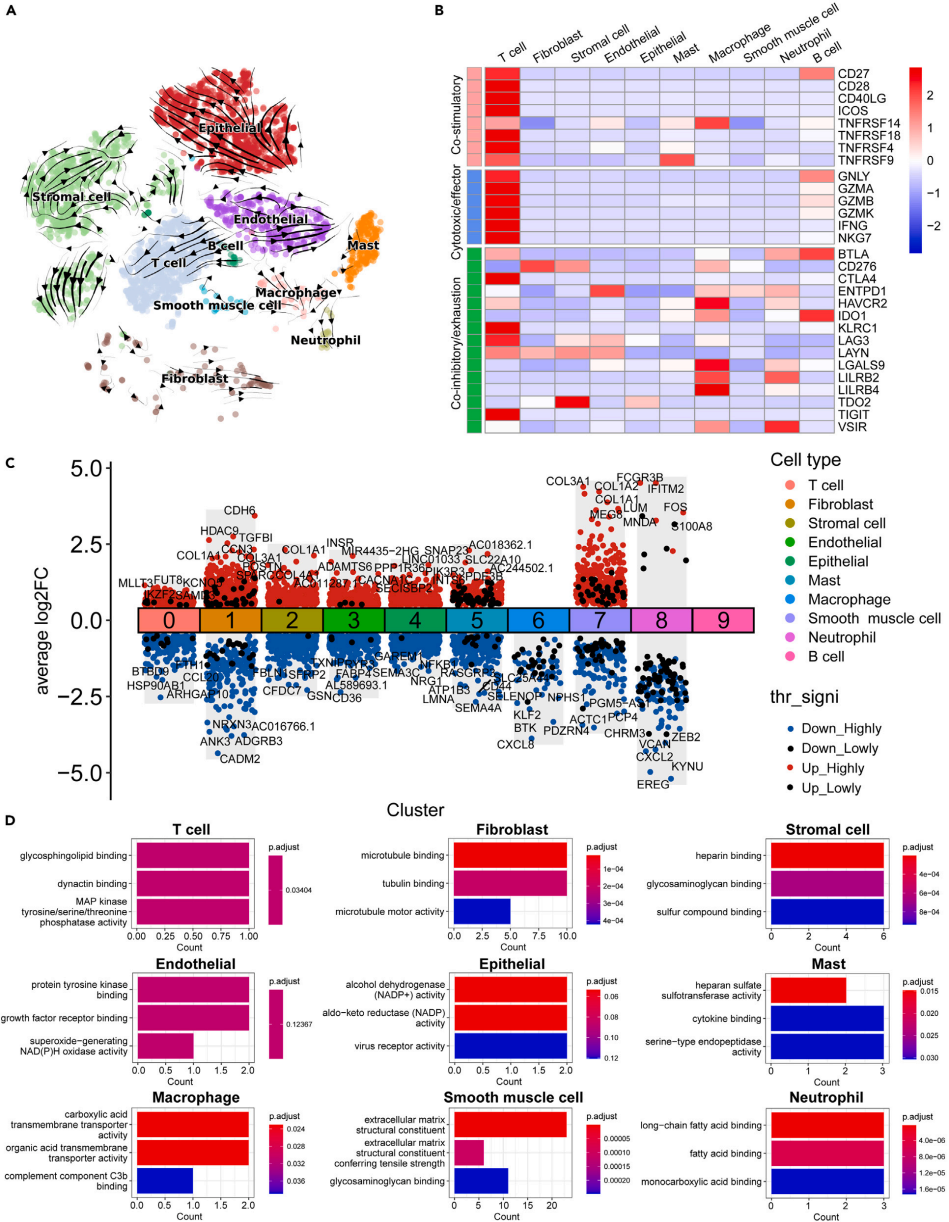
Heterogeneity exists between different cell types (A) Unsupervised pseudotime trajectory of all cell subtypes by RNA velocity. Arrowhead direction represents the trend of cell pseudo-temporal differentiation. (B) Heatmap showing the expression of gene sets associated with different immune functions (co-stimulatory, cytotoxic/effector, and co-inhibitory/exhaustion) in cell types identified by scRNA-seq data. (C) Differential expression analysis revealed differential genes among various cell subtypes. (D) GO enrichment analysis of differential genes in various cell subtypes.

Initially, RNA velocity was utilized to evaluate transcriptional diversity along inferred activation pathways in all cell subtypes. Unsupervised pseudotime trajectories were plotted for each cell subtype based on RNA velocity, revealing inconsistent differentiation paths for individual cell types (Figure 3A). Subsequently, the gene expression profiles related to various immune functions were explored across all cell subtypes (Figure 3B). The findings revealed high expression of co-stimulatory genes (CD27, CD28, CD40LG, ICOS, TNFRSF14, TNFRSF18, TNFSRF4, and TNFRSF9) and cytotoxic/effector genes (GNLY, GZMA, GZMB, GZMK, IFNG, and NKG7) in T cells. Mast cells showed high expression of TNFRSF9, macrophages of TNFRSF14, and B cells of CD27 (Figure 3B). Moreover, concerning co-inhibitory/exhaustion genes, T cells exhibited high expression of CTLA4, KLRC1, LAG3, and TIGIT, B cells of BTLA and IDO1, and macrophages of HAVCR2, LGALS9, LILRB2, and LILRB4 (Figure 3B). Lastly, differentially expressed genes in distinct cell subtypes and presented as the results of GO enrichment analysis (Figure 3C). The results highlighted enrichment patterns such as microtubule binding in fibroblasts, alcohol dehydrogenase (NADP+) activity in epithelial cells, and heparan sulfate sulfotransferase activity in mast cells (Figure 3D). These outcomes collectively emphasize the heterogeneity among different cell types.

### ST characterization of bladder ES/PNET and BLCA

**Figure 4:**
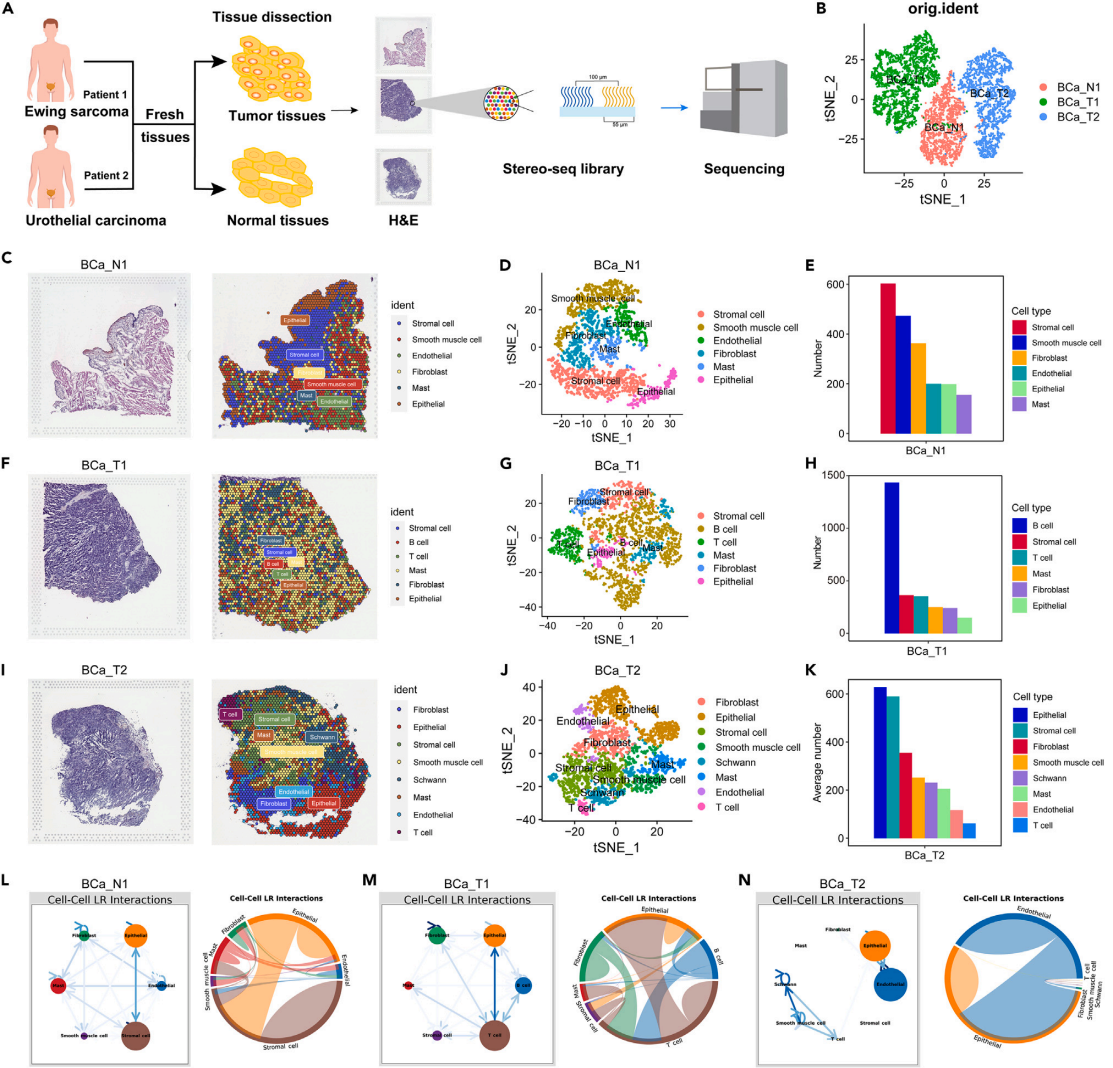
ST characterization of bladder ES/PNET and BLCA (A) Graphic overview of the design diagram for ST. (B) ST plots visualized by t-SNE from three samples. (C) H&E and ST plots of BCa_N1. (D) t-SNE plots of the major 6 cell types in BCa_N1. (E) Number of cells in BCa_N1 for 6 cell types. (F) H&E and ST plots of BCa_T1. (G) t-SNE plots of the major 6 cell types in BCa_T1. (H) Number of cells in BCa_T1 for 6 cell types. (I) H&E and ST plots of BCa_T2. (J) t-SNE plots of the major 8 cell types in BCa_T2. (K) Number of cells in BCa_T2 for 8 cell types. (L) CCI maps of the main 6 cell types in BCa_N1 at ST level. (M) CCI maps of the main 6 cell types in BCa_T1 at ST level. (N) CCI maps of the main 8 cell types in BCa_T2 at ST level.

As previously discussed, scRNA-seq data lack spatial information for individual cells. To address this limitation, ST was employed to enhance the completeness of the sequencing data and uncover the spatial distribution of all cells. Frozen sections of fresh tumor and normal samples were stained with H&E, and bright-field images were captured for subsequent ST sequencing (Figure 4A). Using robust cell type decomposition (RCTD) software, each tissue coverage point was deconvoluted to construct ST maps visualized by t-SNE (Figure 4B). ST analysis revealed the cellular composition within different samples. For instance, BCa_N1 was characterized by the presence of multiple cell types including stromal cells, smooth muscle cells, endothelial cells, fibroblasts, mast cells, and epithelial cells. Among these, stromal cells emerged as the predominant cell type (Figures 4C–4E). BCa_T1 (bladder ES/PNET) displayed a composition comprising stromal cells, B cells, T cells, mast cells, fibroblasts, and epithelial cells, with the highest proportion being B cells (Figures 4F–4H). BCa_T2 (BLCA) exhibited a cellular makeup consisting of fibroblasts, epithelial cells, stromal cells, smooth muscle cells, Schwann cells, mast cells, endothelial cells, and T cells, where epithelial cells were the prevailing cell type (Figures 4I–4K). Additionally, ST analysis was performed, and the cellular composition inference (CCI) map was established. The findings revealed that in BCa_N1 and BCa_T1, epithelial cells exhibited correlations with the other five cell types (Figures 4L and 4M), whereas, in BCa_T2, epithelial cells seemed to correlate primarily with T cells and endothelial cells (Figure 4N). In summary, distinct spatial variances were observed between bladder ES/PNET and BLCA.

### Identification of specific types of epithelial cells (bladder ES-Epi) in bladder ES/PNET using scRNA-seq data

**Figure 5:**
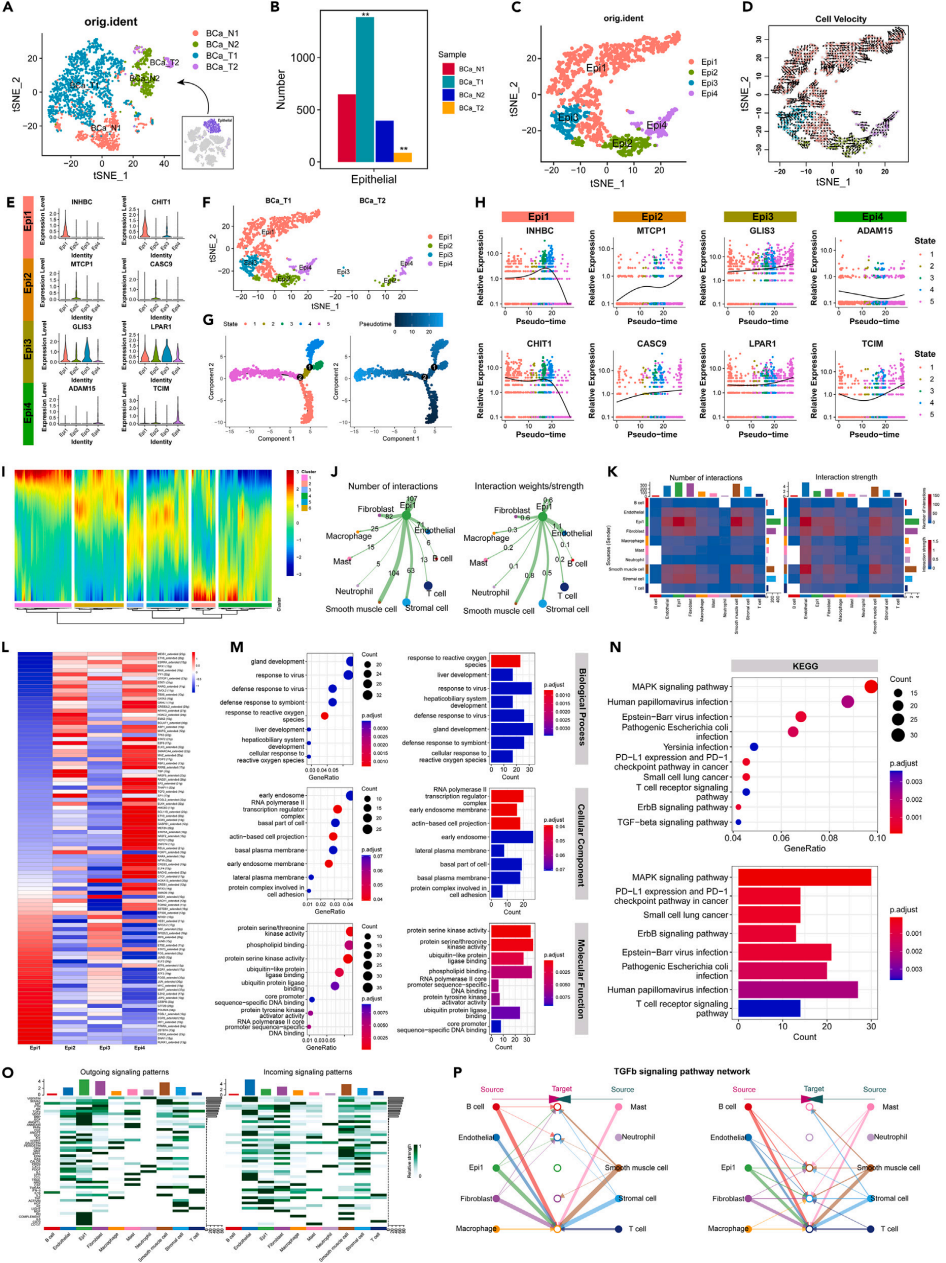
scRNA-seq data identify bladder ES-Epi as a specific type of epithelial cell in bladder ES/PNET (A) t-SNE plots of all epithelial cells in 4 samples. (B) Number of epithelial cells in the 4 samples. (C) Re-annotated t-SNE plots of all epithelial cells in tumor samples. (D) Unsupervised pseudotime trajectory of 4 epithelial cell subtypes by RNA velocity. (E) Expression of marker genes for 4 epithelial cell subtypes. (F) Distribution maps of 4 epithelial cell subtypes in BCa_T1 and BCa_T2. (G) Pseudotime trajectory of 4 epithelial cell subtypes in BCa_T1 and BCa_T2. (H) Smoothed expression curves of representative candidate genes along the trajectory. (I) DEGs between Epi1 (bladder ES-Epi) and the other epithelial cells with pseudotime variation. (J) An overview of Epi1 (bladder ES-Epi) and other cells interactions. Arrow and edge color indicate direction. Circle size is proportional to the number of cells in each cell group. Edge thickness indicates the number and the strength of interaction between populations. (K) Heatmaps of differential number and strength of intercellular interactions between Epi1 (bladder ES-Epi) and other cells. (L) Transcription factor analysis of 4 epithelial cell subtypes. (M) GO analysis of Epi1 (bladder ES-Epi) cell subtype. (N) KEGG analysis of Epi1 (bladder ES-Epi) cell subtypes. (O) Outgoing and incoming signaling patterns of intercellular interactions between Epi1 (bladder ES-Epi) and other cells. (P) Outgoing and incoming signaling patterns of intercellular interactions between Epi1 (bladder ES-Epi) and other cells in TGFb signaling pathway network.

Following the identification of various cell types in both the single-cell RNA sequencing (scRNA-seq) and ST datasets, our focus shifted toward analyzing the gene expression trajectory specifically within epithelial cells. Initially, 2,516 epithelial cells were analyzed across four samples (Figure 5A). A notable increase in epithelial cells was observed in BCa_T1, while a decrease was noted in BCa_T2 within the matched tumor and normal tissues (Figure 5B). Subsequently, the epithelial cells from BCa_T1 and BCa_T2 were merged, resulting in a refined dataset of 1,474 epithelial cells (Figure S1). Upon re-clustering this merged scRNA-seq epithelial cell dataset, four distinct subclusters of epithelial cells were successfully identified (Figure 5C) and delineated their unsupervised pseudotime trajectory (Figure 5D). Notably, INHBC and CHIT1 were identified as marker genes for Epi1, MTCP1, and CASC9 for Epi2, GLIS3, and LPAR1 for Epi3, and ADAM15 and TCIM for Epi4 (Figure 5E). Upon further separation of BCa_T1 and BCa_T2, BCa_T1 was expressed in all four epithelial cell subclusters, while BCa_T2 was expressed in only three subclusters (Figures 5F and S2). This finding suggests that Epi1 represents a distinct type of epithelial cell found specifically in BCa_T1, defining it as bladder ES-Epi. Pseudotime analysis of all epithelial cells indicated that bladder ES-Epi occupied one end of the trajectory (Figure 5G). Finally, the smooth expression profiles along the trajectory of the four epithelial cell subcluster marker genes (Figure 5H).

Subsequently, all genes associated with the pseudotime trajectory of bladder ES-Epi were identified, which can be categorized into six modules. Modules 1 and 6 exhibited significant upregulation, while modules 2 and 5 showed significant downregulation (Figure 5I). CCI of the four epithelial cell subclusters with other cells was assessed. The results indicated observed interactions between the four epithelial cell subclusters and other cell groups within BCa_T1 (Figure S3). Moreover, bladder ES-Epi demonstrated heightened interactions, displaying the strongest association with smooth muscle cells in BCa_T1 (Figure 5J). Communication probability results revealed that bladder ES-Epi cells had greater interactions with fibroblasts and smooth muscle cells, while their interaction with endothelial cells was notably robust (Figure 5K).

Single-cell regulatory network inference and clustering (SCENIC) was used to construct a single-cell regulatory network, identifying potential key TFs for bladder ES-Epi, including ZBTB7A, EGR3, EZH2, MYC, and JUN (Figure 5L). Subsequent GO analysis disclosed significant enrichment in biological processes related to reactive oxygen species response and gland development for bladder ES-Epi. Additionally, enrichment in cellular components was observed in RNA polymerase II transcription regulator complex and early endosome, while molecular functions were enriched in protein serine/threonine kinase activity (Figure 5M). Furthermore, Kyoto Encyclopedia of Genes and Genomes (KEGG) pathway analysis indicated a significant enrichment of the MAPK signaling pathway (Figure 5N). Further, by examining outgoing or incoming signaling patterns, heightened activity in the tumor growth factor b (TGF-b) signaling pathway was discovered across all signaling patterns (Figure 5O). Investigation into the outgoing or incoming signaling patterns of the TGF-b pathway revealed that bladder ES-Epi could affect the same targets in collaboration with various cells apart from neutrophils (Figure 5P).

### Specific types of mast cells in bladder ES/PNET (bladder ES-Mast)

**Figure 6:**
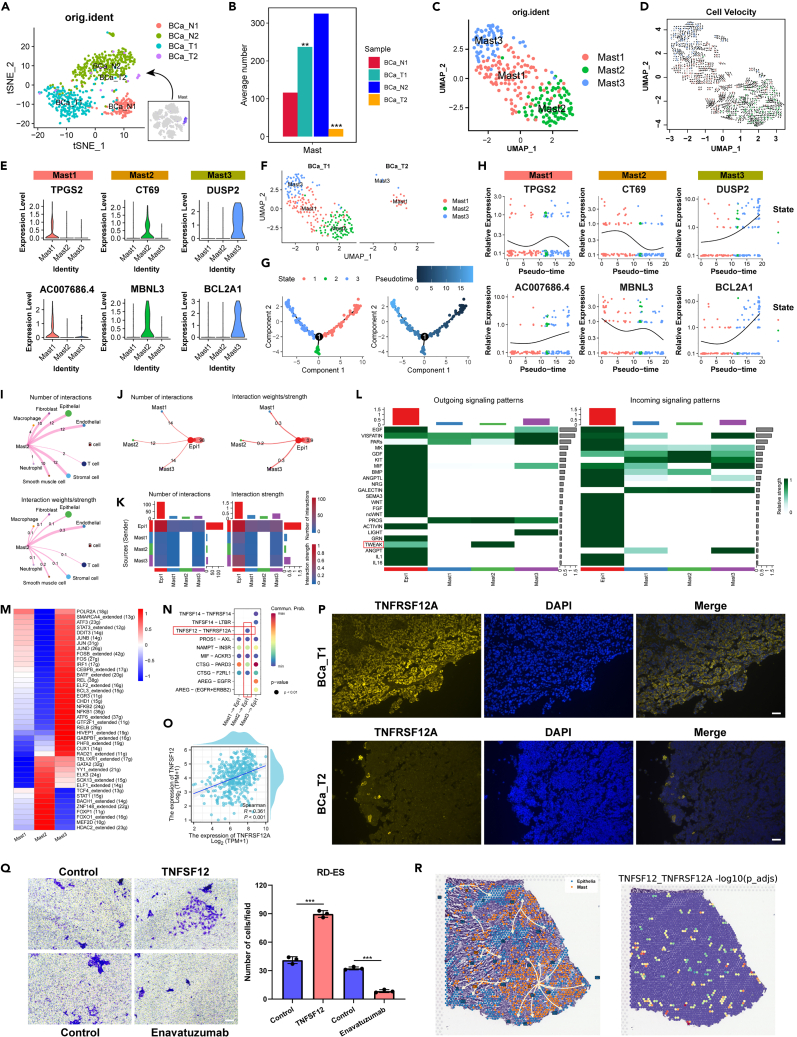
Bladder ES-mast is a specific type of bladder ES/PNET mast cells and promotes bladder ES/PNET progression via TNFSF12-TNFRSF12A ligand-receptor signaling pattern (A) t-SNE plots of all mast cells in 4 samples. (B) Number of mast cells in the 4 samples. (C) Re-annotated t-SNE plots of all mast cells in tumor samples. (D) Unsupervised pseudotime trajectory of 3 mast cell subtypes by RNA velocity. (E) Expression of marker genes for 3 mast cell subtypes. (F) Distribution maps of 3 mast cell subtypes in BCa_T1 and BCa_T2. (G) Pseudotime trajectory of 3 mast cell subtypes in BCa_T1 and BCa_T2. (H) Smoothed expression curves of representative candidate genes along the trajectory. (I) An overview of mast2 (bladder ES-Mast) and other cells interactions. Arrow and edge color indicate direction. Circle size is proportional to the number of cells in each cell group. Edge thickness indicates the number and the strength of interaction between populations. (J) An overview of Epi1 (bladder ES-Epi) and other 3 mast cell subtypes interactions. (K) Heatmaps of differential number and strength of intercellular interactions between Epi1 (bladder ES-Epi) and other 3 mast cell subtypes. (L) Outgoing and incoming signaling patterns of intercellular interactions between Epi1 (bladder ES-Epi) and other 3 mast cell subtypes. (M) Transcription factor analysis of 3 mast cell subtypes. (N) Ligand-receptor pair plot for Epi 1 (bladder ES-Epi) and other 3 mast cells subtypes. (O) Scatterplot of correlation between TNFRSF12A and TNFSF12 in the TCGA-BLCA database. (P) Immunofluorescence staining of TNFRSF12A in BCa_T1 and BCa_T2 (Scale bars, 100 μm). (Q) Effect of exogenous supplementation with TNFSF12 or Enavatuzumab on the migratory capacity of RD-ES cells. (Scale bars, 100 μm, *n* = 3, Data were analyzed with use of Student’s t test, and represented as mean ± SEM, ∗∗∗*p* < 0.001). (R) Localization of epithelial cells and mast cells, as well as TNFRSF12A and TNFSF12 at the ST level.

The analogous alterations in epithelial cells and mast cells in bladder ES/PNET and BLCA tissues led to speculation regarding the crucial role of mast cells in the regulation of epithelial cells (Figure 6A). A bar graph illustrated an increase in mast cells in bladder ES/PNET tumor tissues and a decrease in BLCA tissues (Figure 6B). This study scrutinized 257 mast cells from BCa_T1 and BCa_T2 and re-clustered the merged mast cells, obtaining three mast cell subclusters and plotting their associated unsupervised pseudotime trajectory (Figures 6C, 6D, S4, and S5). Markedly, TPGS2 and AC007686.4 were identified as marker genes for Mast1; CT69 and MBNL3 for Mast2; and DUSP2 and BCL2A1 for Mast3 (Figure 6E). Similarly, upon segregating BCa_T1 and BCa_T2, Mast2 was identified as a specific mast cell type in BCa_T1, termed bladder ES-Mast (Figure 6F). Pseudotime analysis of all mast cells revealed that bladder ES-Mast occupies a distinct segment of the trajectory (Figure 6G). Additionally, the smoothed expression profiles along the trajectory for the marker genes of the three mast cell subclusters are displayed in Figure 6H.

Intercellular communication among three distinct mast cell subclusters and various cell types was investigated to comprehend the broader scenario. The findings highlight significant crosstalk among the three mast cell subclusters and other cell types, excluding B cells (Figures 6I and S6). Notably, interactions were identified between bladder ES-Epi and the three mast cell subclusters (Figures 6J and 6K). Subsequent analysis of outgoing signaling patterns emphasized heightened activity within the TNF-related weak inducer of apoptosis (TWEAK) signaling pathway between bladder ES-Epi and bladder ES-Mast (Figure 6L). Single-cell regulatory network analysis identified HDAC2, FOXO1, and STAT1 as potential key TFs in bladder ES-Mast (Figure 6M). Moreover, GO and KEGG analyses revealed significant enrichment in processes such as regulation of RNA splicing, focal adhesion, GTPase regulator activity, and MAPK signaling pathways (Figure S7).

### CCI analysis revealed TNFSF12-TNFRSF12A ligand-receptor pair activation associated with bladder ES/PNET progression

Given the interaction between bladder ES-Epi and bladder ES-Mast cells, the specific ligand-receptor pairs between these two cell subclusters were further explored. The investigation identified the “TNFSF12-TNFRSF12A” ligand-receptor pair as notably present in bladder ES-Epi→bladder ES-Mast cells compared with the relationships between bladder ES-Epi and the other two mast cell subclusters (Figure 6N). TNFSF12, also known as TNF-related weak inducer of apoptosis (TWEAK), has been reported to stimulate the survival and angiogenesis of urothelial carcinoma through TNFRSF12A/VEGF signals.^19^ Initial assessment of TNFRSF12A expression in BLCA revealed its overexpression in tumors, with higher expression associated with advanced pathological staging and a poorer prognosis (Figure S8). Furthermore, correlation analysis unveiled a positive association between TNFSF12-TNFRSF12A ligand-receptor expression in BLCA (Figure 6O). Subsequent examination of TNFRSF12A expression in bladder ES/PNET and BLCA tissue samples revealed significant overexpression in bladder ES/PNET (Figure 6P). Moreover, ELISA results showed that the expression of TNFSF12 in the urine of bladder ES/PNET patient was higher than that in normal individuals (Figure S9). Additionally, multiple immunofluorescence staining results showed that the tumor-associated mast cell marker gene TPGS2 co-localized with TNFSF12, and the tumor-associated epithelial cell marker gene INHBC co-localized with TNFRSF12A (Figure S10). Enavatuzumab, a humanized anti-TWEAK receptor monoclonal antibody, recruits and activates myeloid effector factors to kill tumor cells and exerts its potent anti-tumor activity.^20^ Functional experiments suggested that exogenous TNFRSF12 significantly enhanced the migration abilities of the ES cell line RD-ES and BLCA cell line T24, as well as the angiogenesis ability of RD-ES, while Enavatuzumab exerts the opposite function (Figures 6Q, S11A, and S11B). Finally, spatial analysis results revealed mutual interactions between mast cells and epithelial cells, with TNFSF12-TNFRSF12A interactions occurring at the spatial level (Figure 6R).

## Discussion

Primary bladder ES/PNET, a rare malignant tumor originating from migrating embryonic cells of the neural crest, predominantly afflicts adults.^21^ To date, only 27 publications have reported 39 cases of bladder ES/PNET. The clinicopathological features are detailed in Table 1. Among the 40 patients (age range: 45 days to 81 years; male-to-female ratio: 21:19; average age: 43 years), the tumor size averaged 6.81 cm. Fifteen patients underwent partial cystectomy, while 14 patients underwent radical cystectomy. Approximately 82.5% (33/40) received adjuvant chemotherapy, primarily VAC (14 patients) or VAC+IE (13 patients) regimens. The survival curve (Figure S12) suggested a median survival time of 18 months among the 34 patients with follow-up data. Bladder ES/PNET is a rare disease that is associated with a poor prognosis, and definitive guidelines for its management and treatment have not been established. Currently, surgical resection combined with adjuvant chemotherapy appears effective. However, reporting and analyzing additional cases are imperative to establish treatment protocols. Investigating the molecular mechanisms of bladder ES/PNET progression holds significant importance.

**Table 1:** Published cases of bladder Ewing’s sarcoma/primitive neuroectodermal tumor (ES/PNET) to date

Age	Sex	Symptoms	Size (cm)	Local	Metastasis	Surgery	Urinary diversion	Adjuvant therapy	Follow-up	Reference
19	M	Hematuria	4.5	Left lateral wall	No	TURBT, radical cystoprostatectomy	Ileal conduit	Chemotherapy (VAC + IE)	Alive, 12 months	Present case
19	F	Hematuria	5.5	Left posterior	No	TURBT	No	Chemotherapy (gemcitabine)	Alive, 24 months	Tan et al., 2023^22^
59	M	Hematuria, dysuria, pain in right renal fossa	4	Right posterolateral wall	Multiple bone	TURBT, nephrostomy	No	Chemotherapy (VAC)	Died at 4 months	Nakdali et al., 2022^23^
45 days	F	Gross hematuria	1.2	Posterior bladder wall	No	TURBT, partial cystectomy	No	Chemotherapy (VAC + IE)	Alive, 18 months	Howe et al., 2021^24^
66	M	Gross hematuria	3.7	Anterior wall	No	Partial cystectomy	No	Chemotherapy (EP)	Died, 12 months	Wu et al., 2021^25^
4	M	Gross hematuria	3.7	Dome of the bladder	Liver, abdominal wall	TURBT, partial cystectomy	No	Chemotherapy (VAC + IE), radiotherapy	Died, 14 months	Baisakh et al., 2020^26^
7	M	Gross hematuria	15	Dome, trigone, and both ureteric orifices	No	TURBT, radical cystectomy	Ileal conduit	Chemotherapy (VAC), radiotherapy	Alive, 5 months	
15	F	Microscopic hematuria	11	Dome, trigone, neck, and both ureteric orifices	Kidney	TURBT, radical cystectomy	Ileal conduit	Chemotherapy (VAC + IE)	Alive, 9 months	
18	F	Microscopic hematuria	3.5	Dome of the bladder	No	TURBT, partial cystectomy	No	Chemotherapy (VAC)	Unknown, ≥5 months	
21	F	Fever, acute urinary retention	4.4	Dome of the bladder	Liver	TURBT, partial cystectomy	No	Chemotherapy (VAC + IE)	Died at 11 months	
21	F	Lower abdominal pain, gross hematuria	7	Left lateral wall	No	Partial cystectomy	No	Chemotherapy (VAC)	Died at 24 months	
30	F	Gross hematuria	4.8	Trigone and neck	No	TURBT, radical cystectomy	Ileal conduit	Chemotherapy (VAC)	Alive, 7 months	
38	M	Fatigue, incontinence, urgency	6	Left lateral wall and dome	No	TURBT, partial cystectomy	No	Chemotherapy (VAC)	Died at 19 months	
59	F	Lymphedema of the legs	9	Dome, trigone, and both ureteric orifices	No	Radical cystectomy	Ileal conduit	Chemotherapy (VAC)	Unknown, ≥11 months	
61	M	Microscopic hematuria	14	Dome, trigone, neck, and both ureteric orifices	No	TURBT, radical cystoprostatectomy	Ileal conduit	Chemotherapy (VAC)	Alive, 24 months	
62	M	Fever, dull aching lower abdomen pain, lump	8.3	Right lateral wall and dome	No	TURBT, radical cystoprostatectomy	Ileal conduit	Chemotherapy (VAC)	Alive, 18 months	
72	M	Renal failure, B/L hydronephrosis	2.9	Left lateral wall	Lung, liver	Radical cystoprostatectomy	B/L nephrostomy	Chemotherapy (VAC + IE), radiotherapy	Died at 19 months	
81	M	Dark urine, U/L hydronephrosis	6.4	Left lateral wall extending to the neck	Lung liver	Partial cystectomy	U/L nephrostomy	Chemotherapy (VAC + IE), radiotherapy	Died at 7 months	
45	F	Frequency, urgency, dysuria	3	Right side	No	TURBT, cystectomy, hysterectomy	Ileal conduit	Chemotherapy (VAC)	Alive, 24 months	Gao et al., 2020^27^
64	M	Abdomen dull pain	6	Left lateral wall	No	Partial cystectomy	No	Pelvic radiotherapy	Alive, 2 weeks	Liu et al., 2020^28^
78	M	Gross hematuria, blood clot, urinary frequency, urgency	6.3	Right posterior	No	TURBT	No	No	Died at 2 months	Zhang et al., 2020^29^
70	M	Hematuria and irritative urinary symptoms	4.4	Posterior bladder wall	No	TURBT, radical cystoprostatectomy	NA	Chemotherapy (VAC), radiotherapy	Alive, 36 months	Parizi et al., 2019^30^
27	F	Frequency, hematuria	10.3	Left anterior and lateral	Pelvic adenopathies	TURBT	No	Chemotherapy (VAC)	Alive, 3 months	Vallonthaiel et al., 2016^31^
30	F	Polyuria, gross hematuria	9.4	Right side	No	TURBT, radical cystectomy	Indiana pouch	Chemotherapy (VAC + IE)	Alive, 14 months	Lam and Shayegan, 2016^32^
38	F	Gross hematuria	4	Right side	No	TURBT, radical cystectomy	Ileal conduit	Chemotherapy (VAC + IE)	Alive, 14 months	Tonyali et al., 2016^33^
10	M	Polyuria, lower abdomen swelling	13.5	Right side	No	Partial cystectomy	No	Chemotherapy (VAC + IE)	Alive, 11 months	Sueyoshi et al., 2014^34^
14	F	Lower abdominal lump and dull pain	15	Posterior bladder wall	No	Partial cystectomy	No	Chemotherapy	Unknown,≥6 months	Rao et al., 2011^35^
57	F	Pelvic pain, dysuria, urinary frequency, microscopic hematuria	3.3	Right bladder vesical floor	No	TURBT	No	Chemotherapy (VAC + IE)	Alive, 27 months	Busato et al., 2011^36^
65	M	Gross hematuria, dysuria	5	Left posterior	Pelvic lymph node, lungs	TURBT, radical cystoprostatectomy	NA	Chemotherapy (VIDE), radiotherapy	Died at 22 months	Okada et al., 2011^37^
74	M	Frequency, dysuria, hematuria	NA	Bladder neck	Pelvic lymph nodes	TURBT	No	Chemotherapy (VAC)	Died at 4 months	Zheng et al., 2011^38^
67	F	Repeated hematuria, fever, hydronephrosis	3	Posterior	No	TURBT, partial cystectomy	No	Chemotherapy	Died at 8 months	Al Meshaan et al., 2009^39^
16	M	Gross hematuria and dysuria	1	Fundus vesicae felleae	No	TURBT	No	Chemotherapy (CDV + IE)	Alive, 24 months	Osone et al., 2007^40^
21	F	Frequency, dysuria, gross hematuria	9	The posterior and right and left sides	No	TURBT, radical cystectomy	Ileal loop diversion	Chemotherapy, imatinib	Alive, 36 months	Lopez-Beltran et al., 2006^41^
72	M	Gross hematuria, oliguria	NA	Multifocal	Frozen pelvis, ileum	Pelvic lymph nodes dissection, ileum resection	Ileum conduit	NA	Unknown, ≥2 months	Ellinger et al., 2006^42^
81	M	Lymphedema, fatigue, urgency of incontinence	NA	NA	Pelvis, retroperitoneum	TURBT	No	No	Died at 2 weeks	Kruger et al., 2003^43^
61	F	Hydronephrosis and renal failure	NA	NA	Lungs	Bladder biopsy	No	NA	Unknown	Colecchia et al., 2002^44^
38	F	Gross hematuria	12	Posterior and right and left	No	TURBT, radical cystectomy	NA	NA	Unknown	Desai, 1998^45^
62	M	Back ache, acute urinary retention	14	NA	Rectum and retroperitoneum	TURBT	No	No	Died at 3 weeks	Mentzel et al., 1998^46^
15	F	Gross hematuria	3	Right anterior lateral	No	TURBT, partial cystectomy	No	Chemotherapy (VAC + IE)	Alive, 18 months	Gousse et al., 1997^47^
21	M	Microscopic hematuria, dysuria	8	Right side	No	TURBT, partial cystectomy	No	Chemotherapy (VAC)	Alive, 18 months	Banerjee et al., 1997^48^

TURBT: transurethral resection of bladder tumor; EP: etoposide + cisplatin; VAC: vincristine, actinomycin D, cyclophosphamide; IE: ifosfamide, etoposide; CDV: cyclophosphamide, pirarubicin, vincristine; VIDE: vincristine, ifosfamide, doxorubicin, etoposide; NA, not available.

Distinct tumor cell subgroup phenotypes assume varied roles in tumor growth, metastatic potential, and treatment response, underscoring the relevance of studying these subgroups in pertinent ecosystems.^49^^,^^50^^,^^51^ scRNA-seq currently facilitates subgroup identification and delineation of their unique biological effects, serving as a valuable tool in understanding disease pathophysiology.^52^^,^^53^ Conversely, the spatial arrangement and interactions of cells, potentially influencing cell states, remain an urgent research focus.^54^ However, scRNA-seq fails to meet this need as the dissociation process disrupts the spatial structure of tissues. ST addresses this limitation by enabling visualization and quantitative analysis of tissue section structure and spatial levels.^54^ Therefore, integrating these techniques allows for a more comprehensive exploration of cell subgroups pivotal to the pathogenesis of bladder ES/PNET.

Numerous studies have elucidated the pivotal role of epithelial cells in various cancers, such as esophageal squamous cell carcinoma, breast cancer, and bladder cancer.^55^^,^^56^^,^^57^ Mounting evidence suggests that the initiation and progression of cancer significantly rely on diverse stromal cell types within the microenvironment.^58^^,^^59^ Recent investigations highlight how the interplay between epithelial cells and different stromal cell populations influences the progression of tumor cells.^60^^,^^61^ For instance, fibroblasts serve as a crucial element in maintaining the equilibrium of normal tissue epithelial cells within the stroma.^62^ Additionally, mast cells exert significant control over innate and adaptive immune responses by releasing pro-inflammatory mediators in response to various stimuli.^63^^,^^64^ Concurrently, mast cells possess the ability to interact with both immune and non-immune elements in the cancer microenvironment, thereby either promoting or constraining tumor growth.^65^^,^^66^ This study has unearthed the capacity of mast cells to regulate epithelial cells through the “TNFSF12-TNFRSF12A” ligand-receptor pairs. These findings offer fresh perspectives on oncogenic mechanisms and potential therapeutic targets for bladder ES/PNET. In addition, Enavatuzumab can significantly inhibit the migratory ability of the Ewing sarcoma cell line RD-ES, suggesting that Enavatuzumab may serve as a therapeutic option for bladder ES/PNET.

In this study, a significant increase in the proportion of mast cells and epithelial cells was observed in bladder ES/PNET tumor tissue compared to BLCA. Two distinct cell types, bladder ES-Epi and bladder ES-Mast, were identified. Through scRNA-seq analysis, a strong correlation between these two cell types was established. ST further revealed the proximity between bladder ES-Mast and bladder ES-Epi. Additionally, the study delved into the relationship between bladder ES-Epi and bladder ES-Mast, pinpointing the specific presence of the “TNFSF12-TNFRSF12A” ligand-receptor pair between these cells. Outgoing signaling patterns demonstrated the activation of the TEWAK signaling pathway between BES-Epi and BES-Mast. Functional experiments demonstrated that exogenous TNFSF12 enhanced the migration and invasive ability of bladder ES/PNET and BLCA. These findings suggest that mast cell-derived TNFSF12 acting on epithelial TNFRSF12A receptors may represent a critical event in bladder ES/PNET tumorigenesis, shedding light on mechanisms of bladder ES/PNET progression and deepening our understanding of mast cell-to-epithelial cell interactions in cancer initiation and development. This evidence led to the hypothesis that the “TNFSF12-TNFRSF12A” ligand-receptor pair could potentially contribute to the heightened malignancy of bladder ES/PNET.

TNFSF12, also known as TWEAK, participates in the interaction with TNFRSF12A (Fn14) to regulate various pathological processes, including cellular proliferation, migration, apoptosis, inflammation, and angiogenesis.^67^ The “TNFSF12-TNFRSF12A” ligand-receptor pair can activate multiple cell signaling cascades through the nuclear factor-kappa B (NF-κB) and MAPK signaling pathways.^68^^,^^69^ TNFSF12 contributes to renal inflammation; modulates the secretion of chemokines from renal cells; and regulates renal cell proliferation, apoptosis, and differentiation.^70^^,^^71^ Cordido et al.^72^ discovered that the TNFSF12/Fn14 axis contributes to the progression of autosomal dominant polycystic kidney disease. Anti-TNFSF12 reduced the MAPK signaling associated with cystogenesis and cell proliferation and dampened the activation of the NF-κB pathway. The “TNFSF12-TNFRSF12A” ligand-receptor pair is recognized as a key signaling pathway associated with various diseases. Some preclinical models targeting TNFSF12 for treatment have shown promise and ongoing clinical trials are investigating its potential.^71^

In summary, this study underscores the significance of the “TNFSF12-TNFRSF12A” ligand-receptor pair in bladder ES/PNET progression, elucidating the interconnections between mast cells and epithelial cells at single-cell and spatial levels. TNFSF12 secreted by bladder ES-Mast cells activates TNFRSF12A in bladder ES-Epi cells, further promoting the progression of bladder ES/PNET. These findings offer crucial insights into managing and treating bladder ES/PNET by revealing potential underlying mechanisms driving its progression.

### Limitations of the study

In this study, we performed scRNA-seq and ST sequencing of tumor tissues and normal tissues from patients with bladder ES/PNET and BLCA. There are still some limitations of our study. Firstly, we found differences in cell types between the results of scRNA-seq and ST sequencing, which may be due to differences in tissue sampling. Secondly, the rarity of bladder ES/PNET patients requires us to expand the sample for further validation. Thirdly, we did not perform *in vivo* validation of the effect of the TNFSF12-TNFRSF12A ligand-receptor pair. Finally, the therapeutic efficacy of Enavatuzumab in patients with bladder ES/PNET also needs further evaluation.

## Resource availability

### Lead contact

Further information and requests for resources and reagents should be directed to and will be fulfilled by the lead contact, Bin Xu (njxbseu@seu.edu.cn).

### Materials availability

This study did not generate new unique reagents.

### Data and code availability

• All sequencing data in this paper have been deposited at GEO (GSE250523) and are publicly available as of the date of publication. Accession numbers are listed in the key resources table.
• This paper does not report original code.
• Any additional information required to reanalyze the data reported in this paper is available from the lead contact upon request.

## Acknowledgments

This work was supported by 10.13039/501100001809National Natural Science Foundation of China (82403602 to W.-P.M), 10.13039/501100004608Natural Science Foundation of Jiangsu Province (BK20230842 to W.-P.M, BK20231422 to J.-P.W), Research Personnel Cultivation Program of Zhongda Hospital 10.13039/501100008081Southeast University (CZXM-GSP-RC60 to W.-P.M), and Jiangsu Provincial Medical Key Discipline (ZDXK202220 to M.C.). The authors thank Biobank of Zhongda Hospital for providing tissue samples. We also thank Guoqing Ji and Feng Yang from Genechem Biotech Co., Ltd (Shanghai, China) and Chongyi Du from Yilianbo Tech Co., Ltd (Shanghai, China) for their technical support. We also thank Bullet Edits for editing this manuscript.

## Author contributions

W.-P.M., M.C., J.-P.W., S.-Q.C., C.S., and B.X. designed the research. W.-P.M., J.G., S.S., C.-M.G., A.B., C.F., and T.T. performed the research and analyzed results. W.-P.M., K.-J.X., K.-Y.W., H.-L.Z., and J.J. wrote the paper. All authors read and approved the final manuscript.

## Declaration of interests

The authors have declared that no conflict of interest exists.

## STAR★Methods

### Key resources table

**Table:** 

REAGENT or RESOURCE	SOURCE	IDENTIFIER
**Antibodies**		

anti-CLDN1	Abcam	ab307692: RRID:AB_3083082
anti-KRT19	Abcam	ab52625; RRID:AB_2281020
anti-TNFRSF12A	Huabio	ET1611-93; RRID:AB_3070074
anti-TNFSF12	Abcam	ab37170; RRID:AB_778691
anti-TPGS2	Santa cruze	sc-514306; RRID:AB_3661745
anti-INHBC	Huabio	ER63761; RRID:AB_3661744

**Biological samples**		

Fresh primary bladder tumor and paired normal tissue surgical specimens	Affiliated Zhongda Hospital of Southeast University	N/A

**Chemicals, peptides, and recombinant proteins**		

RPMI-1640 medium	Gibco	11875093
Fetal bovine serum	Gibco	10099–141
TWEAK/TNFSF12 protein	MCE	HY-P7309
Enavatuzumab	MCE	HY-P99361
EWSR1 FISH probe	GenePharma	2313
FLI1 FISH probe	GenePharma	2130
TNFSF12 ELISA Kit	Ruixin Biotech	RX2D168306

**Deposited data**		

Raw and analyzed data	This paper	GSE250523

**Experimental models: Cell lines**		

RD-ES	Shanghai Anwei Biotech	AW-CELLS-H0804
T24	Shanghai Anwei Biotech	AW-CELLS-H0363

**Software and algorithms**		

R	https://cran.r-project.org/	Version 4.1.1
SCENIC	https://github.com/aertslab/SCENIC	Version 1.1.2–01
ggplot2	https://cran.r-project.org/	Version 3.3.5
CellChat	https://github.com/sqjin/CellChat	Version 1.0.0
GraphPad Prism	https://www.graphpad.com/features	version 9.0

### Experimental model and study participant details

#### Clinical sample collection

This study included two male patients who underwent radical cystectomy at the Department of Urology, Affiliated Zhongda Hospital of Southeast University. Clinical data for these patients are outlined in Table S1. Follow-up was conducted until November 2023. Fresh primary tumor and paired normal tissue surgical specimens were collected for scRNA-seq and ST sequencing, respectively. The pathologist confirmed the tumor type using paraffin-embedded sections before and after surgery. Preoperative, 1-month postoperative, and 6-month postoperative scans were performed using a 3.0 T magnetic resonance imaging system (MRI; Philips Ingenia II Medical Systems, The Netherlands). The tumor type was confirmed by the pathologist through paraffin sections before and after surgery. The study received approval from the Ethics Committee of Affiliated Zhongda Hospital of Southeast University (2022ZDKYSB155), and written informed consent was obtained from either the patients or their relatives. The methodology adhered to the principles outlined in the Declaration of Helsinki.

#### Cell lines and cell culture

Ewing sarcoma cell line RD-ES and BLCA cell line T24 were purchased from Anwei Biotech (Shanghai, China). Both cell lines were cultured in RPMI-1640 medium (Gibco; USA) supplemented with 10% fetal bovine serum (FBS, Gibco; USA) and 1% penicillin/streptomycin (Gibco; USA), and the cells were cultured in a humidified incubator at 37°C with 5% CO2. The cell lines were stored at −80°C using CELLSAVING reagent (NCM, Suzhou, China).

### Method details

#### Laboratory tests

Blood samples were collected in the morning under fasting conditions, and all analyses were conducted in the Department of Laboratory Medicine. The Coulter DxH 800 analyzer (Beckman Coulter, USA) was utilized, following the manufacturer’s instructions, to assess changes in blood routine parameters such as white blood cell count, red blood cell count, hemoglobin, platelet count, neutrophil count, and lymphocyte count. Additionally, UniCel DxC 800 Synchron (Beckman Coulter, USA) was employed to evaluate changes in liver and kidney function parameters, including total protein, albumin, alanine aminotransferase, aspartate aminotransferase, urea nitrogen, and serum creatinine.

#### Single-cell suspension preparation

Tissue samples (both tumor and normal tissue samples) were excised by an experienced pathologist. Fresh tissue samples were washed three times with saline and divided into two portions for scRNA-seq and ST sequencing. For scRNA-seq, the samples were cut into 1-mm^3^-sized particles using scissors and immersed in a tissue digestion solution comprising trypsin, collagenase type I, collagenase type IV, and hyaluronic acid (all four purchased from Sigma-Aldrich, USA). After digestion at 37°C for 30 min in an oscillating water bath, the mixtures were filtered using a 40-μm filter and centrifuged at 1500 rpm for 5 min to obtain a single-cell suspension. The cells were resuspended in 5 mL of erythrocyte lysate (Sigma-Aldrich, USA), followed by incubation for 3 min and then centrifugation at 1500 rpm for 10 min. Subsequently, the supernatant was removed, and the cells were resuspended in calcium- and magnesium-free phosphate-buffered saline (PBS). Cell count was performed using a fully automated cell counter, and cell viability was assessed.

#### Single-cell RNA-sequencing

The cell suspension concentration was adjusted to 1000 cells/μL. Single-cell suspensions were loaded onto a chromium microfluidic chip using the chromium controller by 10× Genomics to create single-cell gel bead emulsions. Single-cell cDNA libraries were prepared using Chromium Next GEM Single Cell 3ʹ Reagent Kits v3.1 (10× Genomics, Pleasanton, USA) following the manufacturer’s instructions. Sequencing was conducted using Illumina NovaSeq 600 (GeneChem, China) Sequencing Platform. The Cell Ranger Analysis Pipeline (version 6.0.2) was utilized to construct sequencing libraries from single-cell transcriptomes. Reads were mapped to genomes and transcriptomes using the STAR comparator. The "NormalizeData" function in Seurat was used to normalize the count data in the given assay, generating normalized summary data across samples, and generating a matrix of gene counts versus cells.

#### Data preprocessing and quality control

The original BCL files were processed using Illumina’s bcl2fastq converter to obtain raw data from the fastq files. Subsequently, data underwent primary quality control filtering to obtain high-quality clean reads. The filtering criteria were as follows: (a) removal of reads containing polyA; (b) elimination of reads with more than three occurrences; (c) exclusion of low-quality reads (bases with quality value ≤5 accounting for more than 20% of the entire read). Following this, 10× Genomics' official software Cell Ranger was used for cell quality control, detection, and comparison with the reference genome (GRCh38) to generate the original expression matrix.

Subsequent downstream analysis was performed using the Seurat R package. The data underwent further quality control with the following filtering criteria: (a) removal of cells detecting fewer than 200 genes; (b) elimination of cells where the proportion of mitochondrial genes exceeded 20%. After quality control, a total of 15,037 cells were retained for downstream analysis.

#### Batch effect correction and dimensionality reduction

Illumina analysis was conducted to generate single-cell gene expression matrices, and unique molecular identifiers (UMIs) were assigned to each cell. The UMI count data were normalized, screened for highly variable genes (HVGs), and normalized using the SCTransform function. Subsequently, steps were taken in the Seurat package to integrate samples, removing potential batch effects through four steps: selecting genes for integration (SelectIntegrationFeatures function), preparing for integration (PrepSCTIntegration function), finding anchor genes (FindIntegrationAnchors function), and integrating samples (IntegrateData function). Following integration, principal component analysis (PCA) was applied for dimensionality reduction. The ElbowPlot function from the Seurat package was used to visualize the decrease in principle component (PC) variance, and the top 30 PCs were selected for downstream analysis. All cells were sequentially clustered using the FindNeighbors (dims = 1:15) and FindClusters (resolution = 1.2) functions. Two-dimensional visualization of the PCA results was achieved through uniform manifold approximation and projection. Cell clusters were identified using the FindCluster function with resolution set to 1.2. These steps revealed a total of 28 cell clusters.

#### Cell type annotation

Regarding the results of cell clustering, cell types were annotated by analyzing the expression of marker genes using references from the PanglaoDB database (https://panglaodb.se/), CellMarker database (http://biocc.hrbmu.edu.cn/CellMarker/), and related reported marker genes in the literature for different cell types, including T cells marked by CD3D, TRAC, and TRBC2, fibroblasts marked by VIM, stromal cells marked by THY1, LUM, and COL6A3, endothelial cells marked by PECAM1, CDH15, and FLT1, epithelial cells marked by KRT8, KRT19, and CDH1, mast cells marked by MS4A2, macrophages marked by C1QA and C1QB, smooth muscle cells marked by MYH11 and TPM1, neutrophils marked by S100A8 and S100A9, and B cells marked by MS4A1. A total of 10 cell types were identified, and a heatmap was used to visually display the expression of marker genes for each cell type.

#### Differential gene analysis

Differentially expressed genes between subtypes were identified using Seurat’s FindMarkers function with default parameters (*p*-value <0.05 and |log2foldchange| >0.5). These genes were analyzed for Gene Ontology (GO) and Kyoto Encyclopedia of Genes and Genomes (KEGG) pathway enrichment using the clusterProfiler package.

#### Cell trajectory analysis

A proposed temporal analysis of cells was performed using the Monocle2 package. Monocle applies a strategy of sorting individual cells in pseudotime, leveraging asynchronous processes of individual cells to place them on trajectories corresponding to biological processes such as cell differentiation. In this study, the VariableFeatures function in Seurat identified the 1,000 genes with the most significant expression changes between cells. Subsequently, differentiation trajectories based on these 1,000 genes were constructed. The Monocle2 package’s differentialGeneTest function was then applied to infer the set of genes whose expression varied with pseudotime. KEGG signaling pathways enriched by these gene sets were separately analyzed using the clusterProfiler package.

#### Transcription factor regulation analysis

To determine the key regulators dictating epithelial cell and mast cell fate, including transcription factors (TFs) and their target genes, SCENIC software was utilized. This allowed for an analysis of regulatory networks and activities within the cellular system. The AUCell module of SCENIC facilitated the evaluation of transcript regulatory activity.

#### Cellular communication analysis

The interaction between epithelial cells and neurons within ACP tissues at a molecular level was examined using CellChat, a Python-based computational tool. The receptor-ligand pairs meeting specific criteria were selected, which are as follows: either the receptor or ligand was expressed in a minimum of 10% of cells within a cell type, with a *p*-value of less than 0.05, for visualization purposes.

#### ST sequencing analysis

Similar to scRNA-seq, the Illumina bcl2fastq converter was employed to transform BCL files into raw sequencing data, ensuring high-quality clean data post-primary quality control. Primary quality control involved several criteria, which are as follows: (a) removal of read pairs with an N content exceeding 3 in single-end sequencing reads; (b) considering bases with a quality value below 5 as low-quality bases; elimination of read pairs when the number of low-quality bases in single-end sequencing reads exceeded 20% of the length of that read; (d) during adapter sequence removal, the adapter sequence must match at least 8 bp. The obtained clean data were then processed using 10× Genomics' Space Ranger (version 3.1.0) for cell quality control, detection, and alignment to the reference genome (GRCh38), resulting in the raw expression matrix. Downstream analysis of the raw expression matrix involved the utilization of the Seurat package, treating each slice as an independent sample for data filtering, normalization, dimensionality reduction, and visualization. Seurat SCTransform was applied for data standardization, HVG selection, and normalization. PCA was utilized for dimensionality reduction, selecting the top 30 PCs for clustering, with a uniform resolution set at 0.7.

#### Spatial co-localization of receptor-ligand pairs

Spatial co-localization analysis of receptor-ligand pairs was conducted using the stLearn package, integrating known information on receptor-ligand pairing, spatial cell type distribution, and spatial gene expression. Initially, cell type predictions from Seurat were used to annotate the variety of cell types within each spot. Subsequently, essential ligand-receptor pairs between adjacent spots were identified using CellPhoneDB92 implemented in stLearn. Specific receptor-ligand pairs between epithelial and mast cells were then chosen.

#### Spatial cell-to-cell interaction analysis

To explore cell-to-cell interactions (CCI) at spatial locations, known spatial cell type distributions were integrated, and CCI analysis was conducted using the stLearn package. CCI activity at each point was quantified using the "st.tl.cci.merge" function. After reading ST data, low-quality genes were filtered (min_cells = 3), and the ligand-receptor database "connectomeDB2020_lit" was set up to filter out data with min_spots <5.

#### Sample preparation for ST assays

For sample preservation, cleaned and fresh samples were placed in frozen sample embedding boxes containing optimal cutting temperature (OCT; SAKURA) compound. After placement, the box was smoothly transitioned into liquid nitrogen for 30 s to 1 min. Subsequently, the frozen OCT block was wrapped in aluminum foil to shield it from light exposure and placed inside a sealed tape for the subsequent sequencing step.

#### ST sequencing

Sequencing libraries were constructed using Visium spatial gene expression slides and kits according to the manufacturer’s instructions (10× Genomics). The Visium chip comprises 4 capture regions (6.5 × 6.5 mm), each containing approximately 5,000 unique gene spots per area, with individual spots having a diameter of 55 μm and a spot-to-spot center distance of 100 μm. Bright-field images were captured and processed following the protocol (deparaffinization, H&E staining, imaging, and de-crosslinking, CG000409). Spatial gene expression assays were conducted according to protocol CG00407. Library preparation utilized TruSeq Illumina and sequencing was performed on the Illumina NovaSeq 6000 platform, ensuring a minimum sequencing depth of 25,000 reads per tissue coverage point in each capture area. We used the SCTransform method to normalize the ST data, detect highly variable features, and construct a regularized negative binomial model of gene expression.^73^ Additionally, we integrated scRNA-seq and ST data using the "doublet" mode in the robust cell type decomposition (RCTD) deconvolution method.^74^^,^^75^

#### Hematoxylin and eosin staining

Tissue samples were initially fixed in 4% paraformaldehyde, dehydrated using ethanol, and embedded in paraffin. Sections of 5 μm thickness were sliced from paraffin-embedded specimens. These sections were fixed in 95% ethanol for 10–20 min, followed by rinsing with distilled water. Subsequently, the sections were immersed in a hematoxylin staining solution for alkaline staining (5–10 min) and then in an eosin staining solution for acidic staining (5–10 min). Following staining, the sections were rinsed under running water for 1 h and subsequently dehydrated in a graded series of ethanol. Finally, the sections were immersed in xylene for 5 min for transparency. These transparent sections were then blocked with neutral gum, and images were captured using a microscope (Leica Microsystems, Germany).

#### Fluorescence *in situ* hybridization

Fluorescence *in situ* hybridization (FISH) probes targeting EWSR1 and FLI1 were acquired from GenePharma (Shanghai, China) and utilized following the manufacturer’s guidelines. Initially, tissue sections underwent deparaffinization using xylene, followed by dehydration through an ethanol gradient. Post-boiling for 20 min, the sections were air-dried and then treated with proteinase K solution for digestion. Subsequently, the sections were hybridized at 80°C for 5 min and at 37°C overnight after the addition of the FISH probe. The next day, thorough washing was performed with 60% formamide three times for 5 min each, followed by a 10-min wash with 2× sodium citrate saline at room temperature. Finally, cell nuclei were labeled using 4′,6-diamidino-2-phenylindole (DAPI), and high-resolution fluorescence microscopy was employed to capture fluorescence micrographs.

#### Next-generation sequencing

Genomic DNA was isolated from tissue paraffin sections using the ReliaPrep FFPE genomic DNA Miniprep System (Promega, USA) according to the manufacturer’s protocol. Quantification was carried out using the Qubit double-stranded DNA HS Assay Kit (Thermo Fisher Scientific, USA). Subsequently, the DNA extracts were fragmented into 250-bp fragments utilizing the S220 Focusing Ultrasonicator (Covaris, USA), followed by library preparation employing the KAPA Hyper Prep Kit (KAPA Biosystems, USA). The library, indexed accordingly, underwent hybridization with a customized next-generation sequencing (NGS) panel. The Qubit 3.0 Fluorometer (Thermo Fisher Scientific, USA) and LabChip GX Touch HT Analyzer (PerkinElmer, USA) determined the concentration and fragment size distribution of the final libraries, respectively. Sequencing was performed on the NovaSeq 6000 platform (Illumina, USA) for 100 bp paired-end sequencing, and the sequencing results were finally interpreted by 3D Medicines Inc. (Shanghai, China), using Chromas (version 2.33).

#### Immunofluorescence staining

Deparaffinization of paraffin sections occurred initially with water, followed by xylene deparaffinization and dehydration through an ethanol gradient. Sections were boiled for 10 min in Tris-EDTA antigen repair solution, air-dried naturally, and then treated with a 0.3% Triton X-100 solution (Yeasen, Shanghai) for permeabilization. After incubation at room temperature for 25 min, the sections were washed three times with PBS. A drop of 3% hydrogen peroxide methanol solution was added to the sections to block the activity of endogenous catalase. After the sections were allowed to stand at room temperature for 10 min, they were treated with animal-free blocking solution (1×) and incubated at room temperature for 15 min. Primary antibodies were then added to the sections, followed by overnight incubation at 4°C in the dark. The next day, secondary antibodies were added to the sections and incubated at room temperature for 60 min in the dark, followed by PBS washing. Subsequently, the sections were completely covered with fluorescein tyramide working solution and incubated at room temperature for 15 min. Finally, cell nuclei were labeled with DAPI, sections were sealed with an anti-fade mounting medium, and photographs were captured under a microscope. Primary antibodies used were anti-CLDN1 (Abcam, USA), anti-KRT19 (Abcam, USA), anti-TNFRSF12A (Huabio, China), anti- TNFSF12 (Abcam, USA), anti- TPGS2 (Santa cruze, USA), and anti-INHBC (Huabio, China).

#### Transwell migration assays

Cell migration capabilities were evaluated using 8 μm Transwell chambers (Corning, USA). A total of 3 × 105 cells and Enavatuzumab (10 μg/mL) were seeded into the upper chamber, while the lower chamber received 10% fetal bovine serum supplementation. Following 12–24 h of incubation, crystal violet (0.1%; Vicmed, China) was used to fix invaded and migrated cells, and an inverted microscope was used to capture images for counting.

#### Tube formation assay

In a 96-well plate (Corning, USA), 50 μL of Matrigel (BD Biosciences, USA) was added and incubated at 37°C for 1 h. Then, 5000 HUVECs were seeded per well and cultured in a medium containing preconditioned cell supernatant. After 6 h, photographs were taken and tube formations were counted.

### Quantification and statistical analysis

Statistical analyses and graphical representations were conducted using R software (version 4.1.1) and GraphPad Prism software (version 9.0). Experimental data are presented as mean ± standard deviation. Various statistical tests including Student’s t-test, Mann-Whitney U-test, Fisher’s exact test, Wilcoxon rank-sum test, Spearman’s rank test, and log rank test were employed in this study. A *p*-value of <0.05 was considered statistically significant. ∗ represents *p* < 0.05, ∗∗ represents *p* < 0.05, ∗∗∗ represents *p* < 0.001.

## Supplemental information

Document S1. Figures S1‒S12 and Table S1
