# Supplementary material for: Single-cell RNA sequencing and spatial transcriptomics of bladder Ewing sarcoma
Source: iScience. 2024 Sep 11;27(10):110921. doi: 10.1016/j.isci.2024.110921 (PMC11462044; doi:10.1016/j.isci.2024.110921)
Supplement: Document S1. Figures S1‒S12 and Table S1 [file mmc1.pdf]

iScience, Volume *27*

Supplemental information

Single-cell RNA sequencing and spatial transcriptomics of bladder Ewing sarcoma

Weipu Mao, Kangjie Xu, Keyi Wang, Houliang Zhang, Jie Ji, Jiang Geng, Si Sun, Chaoming Gu, Atrayee Bhattacharya, Cheng Fang, Tao Tao, Ming Chen, Jianping Wu, Shuqiu Chen, Chao Sun, and Bin Xu

Supplemental table and figures

Table S1. Characteristics of patients included in this study.

Characteristics of the two patients included in this study for scRNA-seq analysis and spatial transcriptomics research.

Patient number	Pathological type	Age	Gender	TNM stage	Smoking history
T1	Ewing sarcoma	19	Male	T3N0M0	No
T2	Urothelial carcinoma	75	Male	T2bN0M0	No

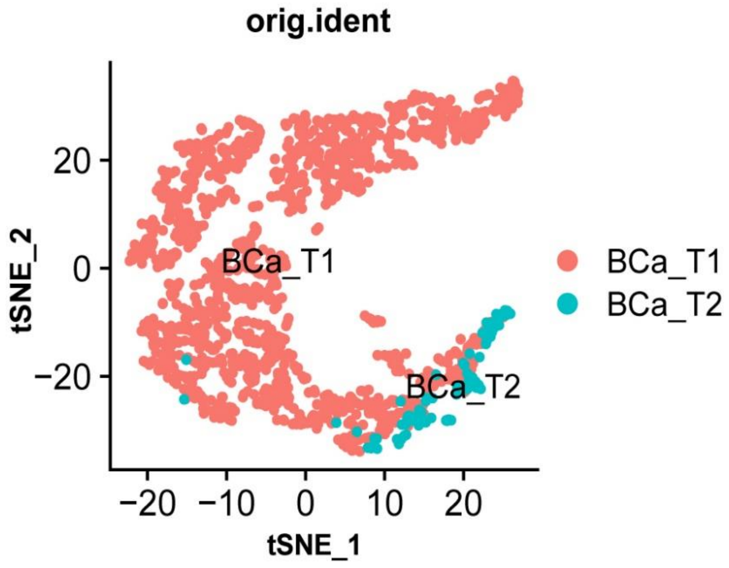

Figure S1. Re-annotated t-SNE plots of epithelial cells in BCa_T1 and BCa_T2, related to Figure 5.

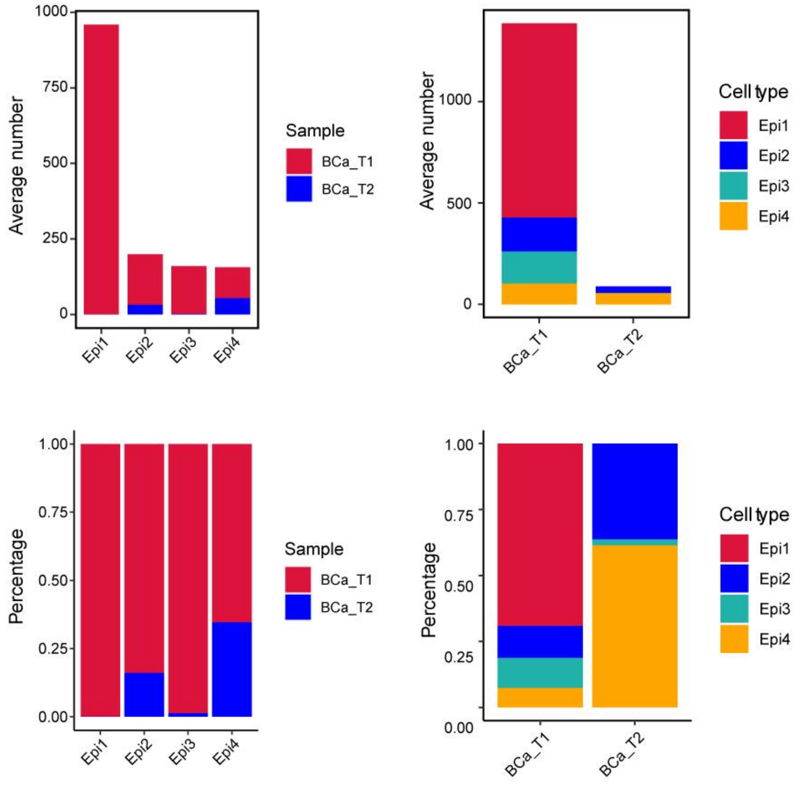

Figure S2. Number and percentage of cells in in BCa_T1 and BCa_T2 for epithelial cell subtypes, related to Figure 5.

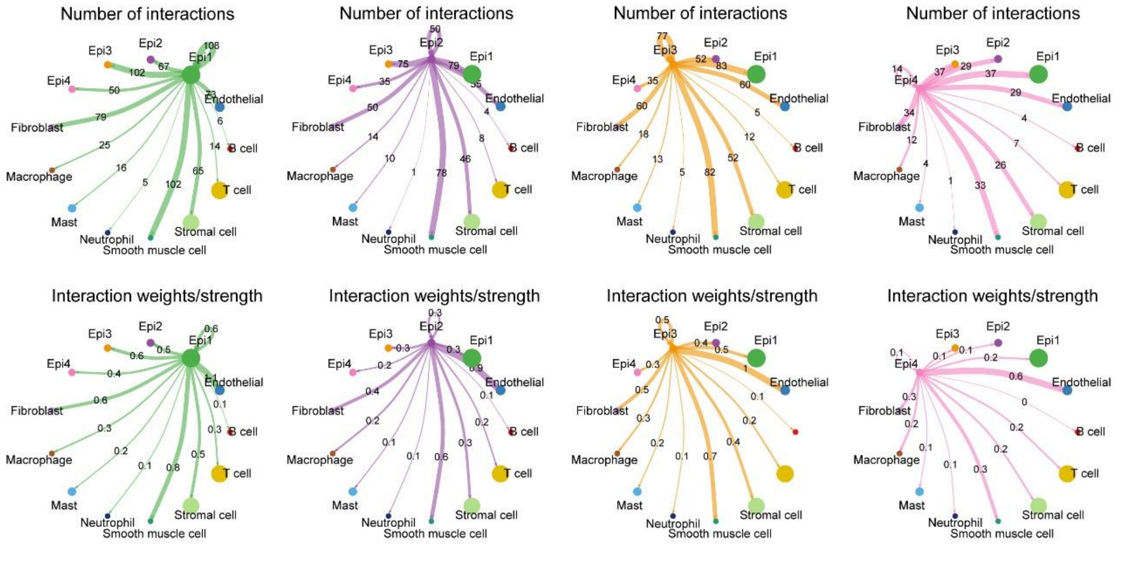

Figure S3. An overview of 4 epithelial cell subtypes and other cells interactions, related to Figure 5.

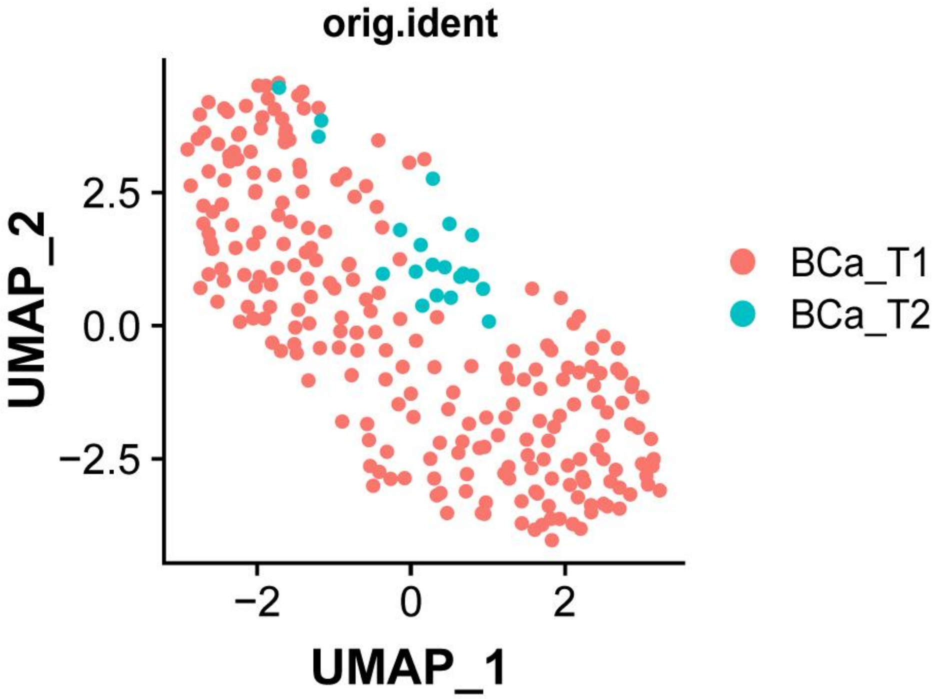

Figure S4. Re-annotated t-SNE plots of mast cells in BCa_T1 and BCa_T2, related to Figure 6.

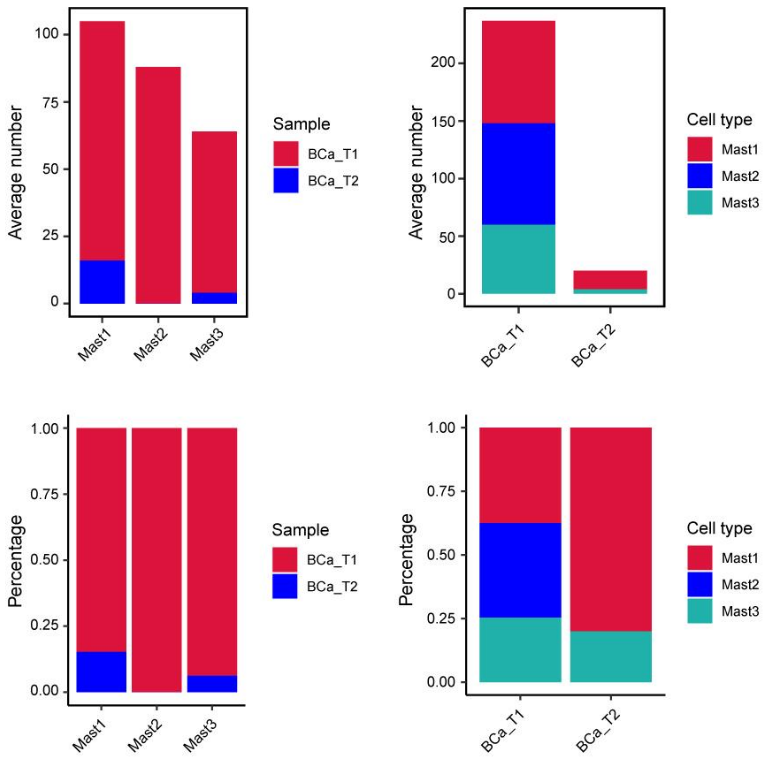

Figure S5. Number and percentage of cells in in BCa_T1 and BCa_T2 for mast cell subtypes, related to Figure 6.

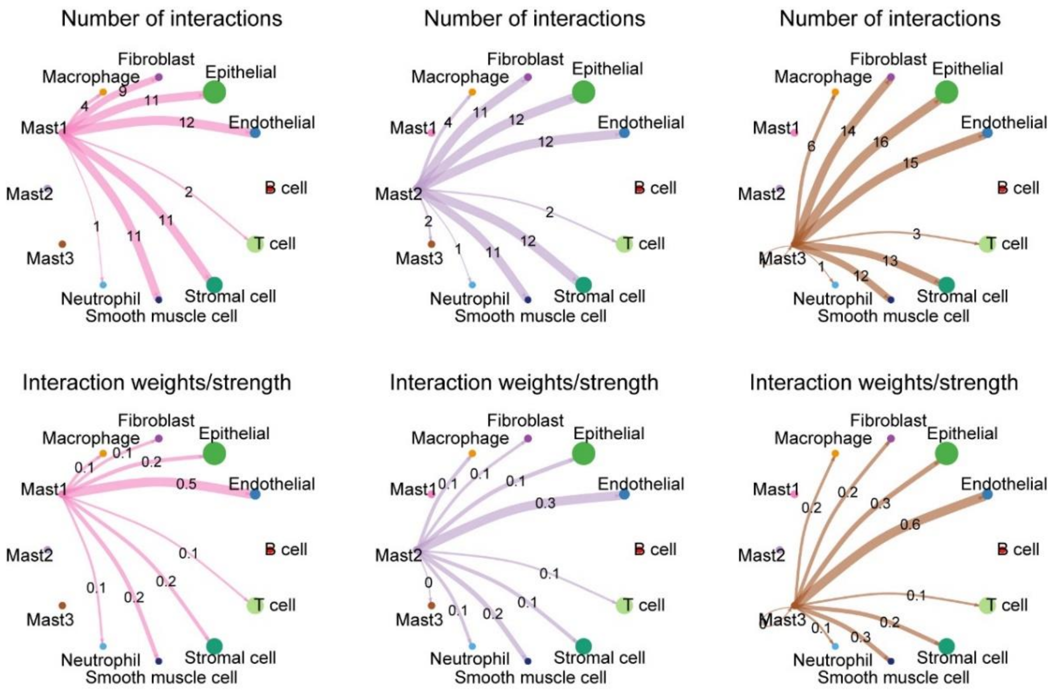

Figure S6. An overview of 3 mast cell subtypes and other cells interactions, related to Figure 6

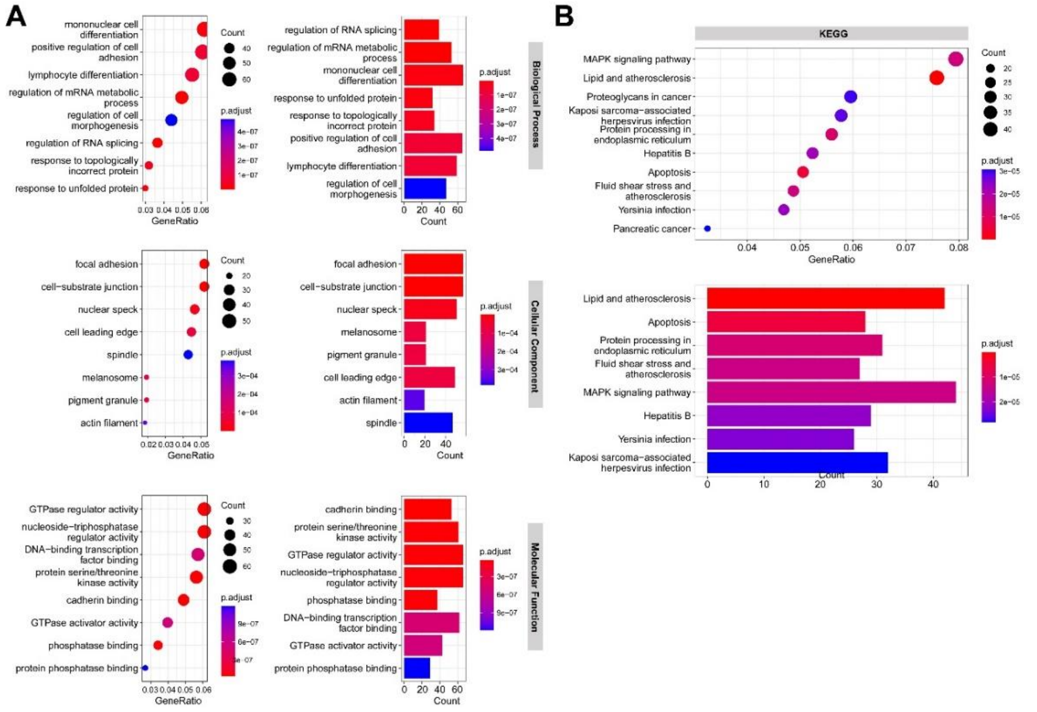

Figure S7. GO analysis and KEGGanalysis of Mast2 (bladder ES-Mast) cell subtypes, related to Figure 6. **A**. GO analysis; **B**. KEGG analysis.

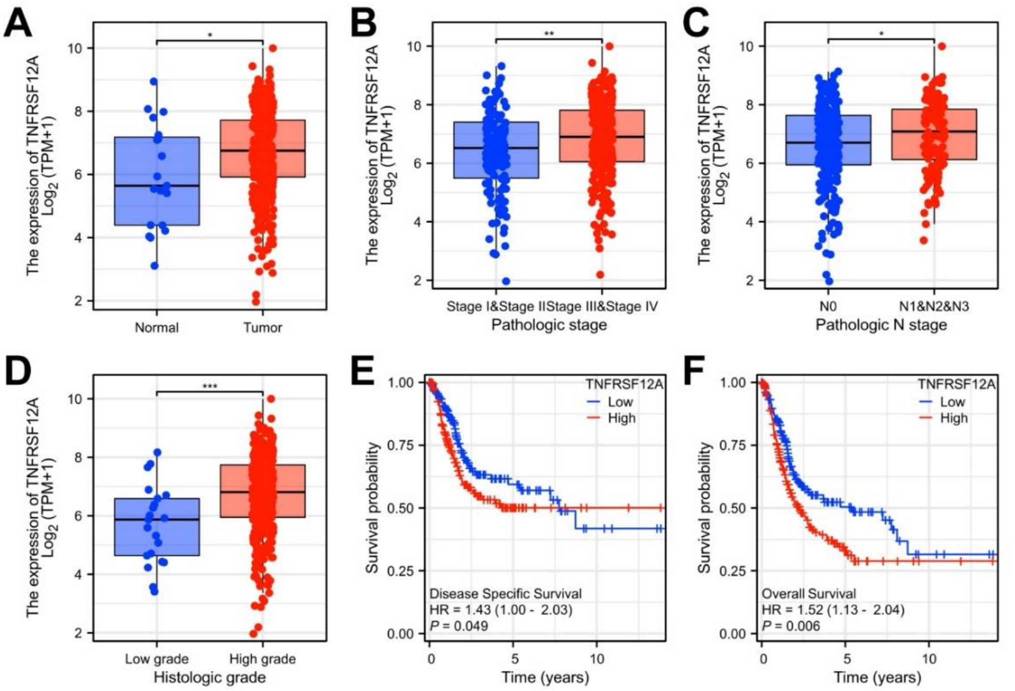

Figure S8. TNFRSF12A expression and prognostic relationship in the TCGA-BLCA database, related to Figure 6. **A**. Normal and tumor; **B**. Pathologic stage; **C**. Pathologic N stage; **D**. Histologic grade; **E**. Disease specific survival curve; **F**. Overall survival curve. (Data were analyzed with use of Student t test, and represented as mean +/- SEM, *p<0.05, **p<0.01, ***p<0.001).

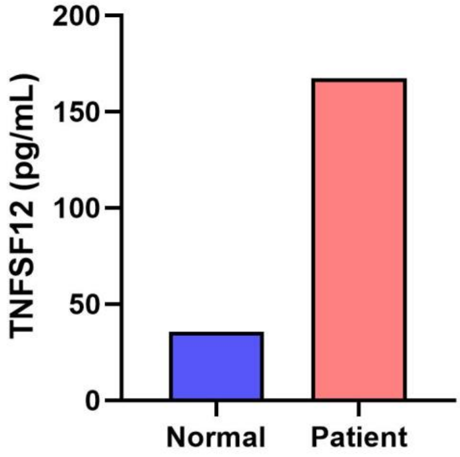

Figure S9. ELISA assay to detect the expression levels of TNFSF12 in the urine of bladder ES/PNET patients and healthy control, related to Figure 6

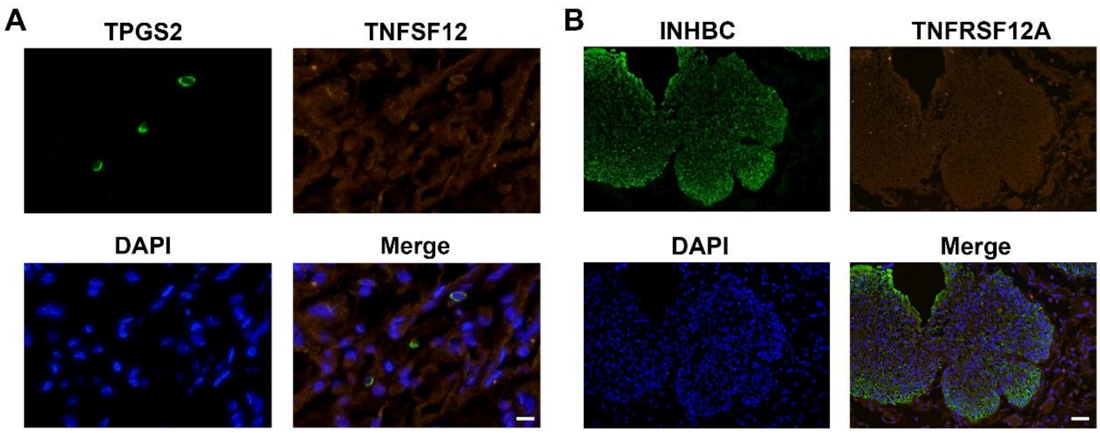

Figure S10. Immunofluorescence staining results, related to Figure 6. **A**. Co-localization of the tumor-associated mast cell marker gene TPGS2 with TNFSF12 (Scale bars = 20 μm); **B**. Co-localization of the tumor-associated epithelial cell marker gene INHBC with TNFRSF12A (Scale bars = 100 μm).

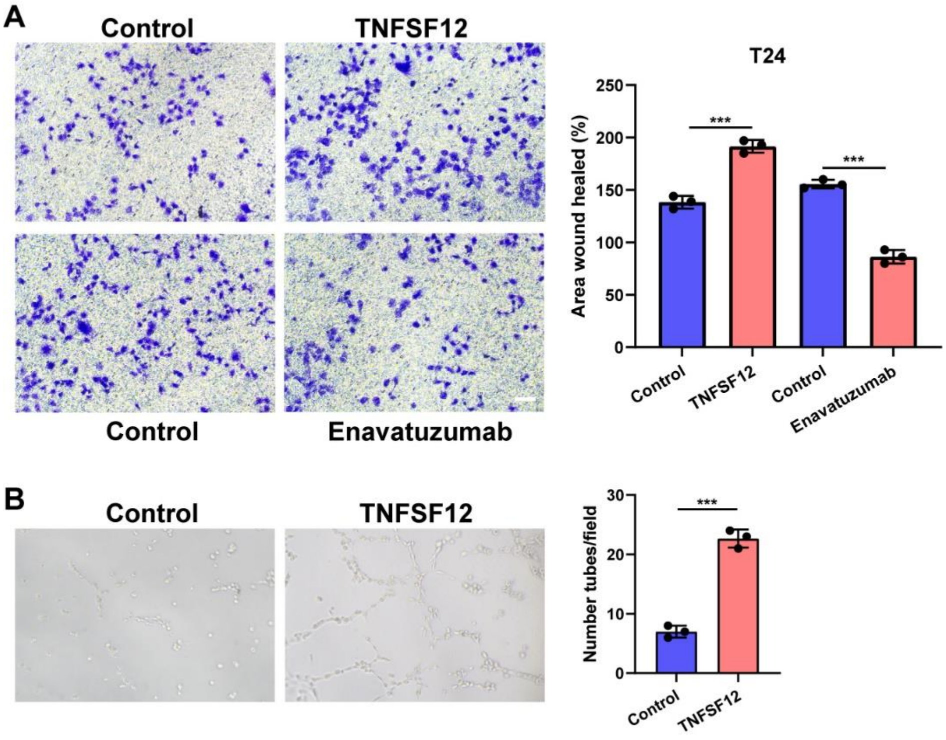

Figure S11. Effect of exogenous supplementation with TNFSF12 or Enavatuzumab on cell migration and cell angiogenesis capacity, related to Figure 6. **A**. T24 cell migration ability; **B**. RD-ES cell angiogenic capacity. (Scale bars = 100 μm, n=3, Data were analyzed with use of Student t test, and represented as mean +/- SEM, ***p<0.001).

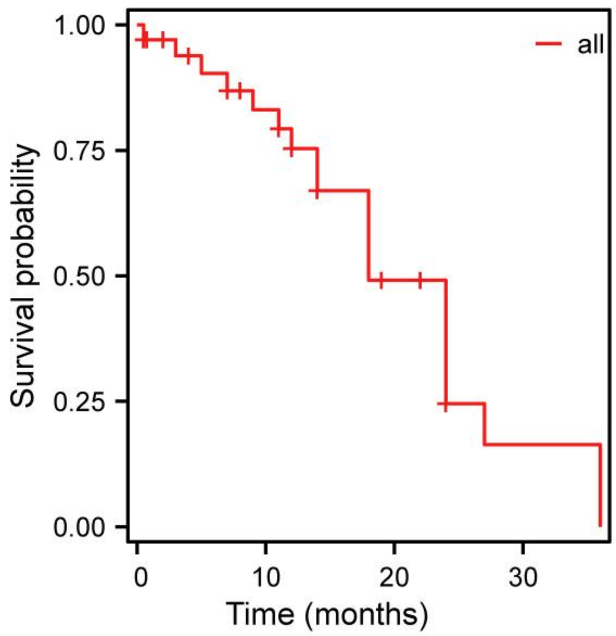

Figure S12. Survival curves in 40 bladder ES/PNET patients.
